# Modeling rheumatoid arthritis using different techniques - a review of model construction and results

**DOI:** 10.1186/s13561-014-0018-2

**Published:** 2014-09-16

**Authors:** Stefan Scholz, Thomas Mittendorf

**Affiliations:** Bielefeld University, Faculty of Public Health, Universitätstr. 25, 33615 Bielefeld, Germany; herescon GmbH, Königsworther Straße, Königsworther Str. 2, 30167 Hannover, Germany

**Keywords:** Rheumatoid arthritis, Modeling, Markov, Discrete-event simulation, TNF-inhibitors, Biologicals, Cost-effectiveness-analysis

## Abstract

**Background:**

Rheumatoid arthritis (RA) is a chronic, inflammatory disease with severe effects on the functional ability of patients. Due to the prevalence of 0.5 to 1.0 percent in western countries, new treatment options are a major concern for decision makers with regard to their budget impact. In this context, cost-effectiveness analyses are a helpful tool to evaluate new treatment options for reimbursement schemes.

**Objectives:**

To analyze and compare decision analytic modeling techniques and to explore their use in RA with regard to their advantages and shortcomings.

**Methods:**

A systematic literature review was conducted in PubMED and 58 studies reporting health economics decision models were analyzed with regard to the modeling technique used.

**Results:**

From the 58 reviewed publications, we found 13 reporting decision tree-analysis, 25 (cohort) Markov models, 13 publications on individual sampling methods (ISM) and seven discrete event simulations (DES). Thereby 26 studies were identified as presenting independently developed models and 32 models as adoptions. The modeling techniques used were found to differ in their complexity and in the number of treatment options compared. Methodological features are presented in the article and a comprehensive overview of the cost-effectiveness estimates is given in Additional files 1 and 2.

**Discussion:**

When compared to the other modeling techniques, ISM and DES have advantages in the coverage of patient heterogeneity and, additionally, DES is capable to model more complex treatment sequences and competing risks in RA-patients. Nevertheless, the availability of sufficient data is necessary to avoid assumptions in ISM and DES exercises, thereby enabling biased results. Due to the different settings, time frames and interventions in the reviewed publications, no direct comparison of modeling techniques was applicable. The results from other indications suggest that incremental cost-effective ratios (ICERs) do not differ significantly between Markov and DES models, but DES is able to report more outcome parameters.

**Conclusions:**

Given a sufficient data supply, DES is the modeling technique of choice when modeling cost-effectiveness in RA. Otherwise transparency on the data inputs is crucial for valid results and to inform decision makers about possible biases. With regard to ICERs, Markov models might provide similar estimates as more advanced modeling techniques.

**Electronic supplementary material:**

The online version of this article (doi:10.1186/s13561-014-0018-2) contains supplementary material, which is available to authorized users.

## Introduction

In most industrialized countries the financial burden of financing health care systems has increased over the last years due to the rise of chronic diseases in consequence of demographic changes as well as the progress in the development of medical technologies. To counteract these developments, regulatory institutions and decision makers have a growing demand for transparent and valid information on the relative value of innovations in comparison to established technologies. Therefore data on relative efficacy as well as effectiveness with respect to specific patient populations today is expected by regulators and payers in the major markets to support the decision on whether to include a new treatment option into reimbursement schemes and treatment guidelines.

In this context different techniques for conducting cost-effectiveness analyses (CEAs) surfaced over the last 15 years, enabling a comparison of (incremental) costs and outcomes of new interventions vs. existing comparators or competitors. Thereby the field of modeling offers a variety of different methods allowing for an estimation of even long-term impacts of certain treatments on the course of a disease beyond the time of clinical studies. [1]. These different approaches might have different pros and cons, e.g. with respect to the method itself and also in specific diseases, which needs to be considered by regulators and decision makers [2].

Over the past 15 years rheumatoid arthritis (RA) has been one of the most competitive disease areas with the introduction of new treatment strategies, e.g. various biologic therapies, leading to a broad body of literature on health economic research. Therefore, the aim of the present review is to explore the field of cost-effectiveness modeling in RA with respect to the different methods used. In this context strengths and weaknesses with respect to appropriate modeling approaches with respect to the course of RA itself as well as a comprehensive provision of treatment strategies are examined. Finally, some recommendations on the interpretation of the results from different modeling types or approaches and potential advantages of certain modeling techniques in future studies are given.

## Methods

**Figure 1:**
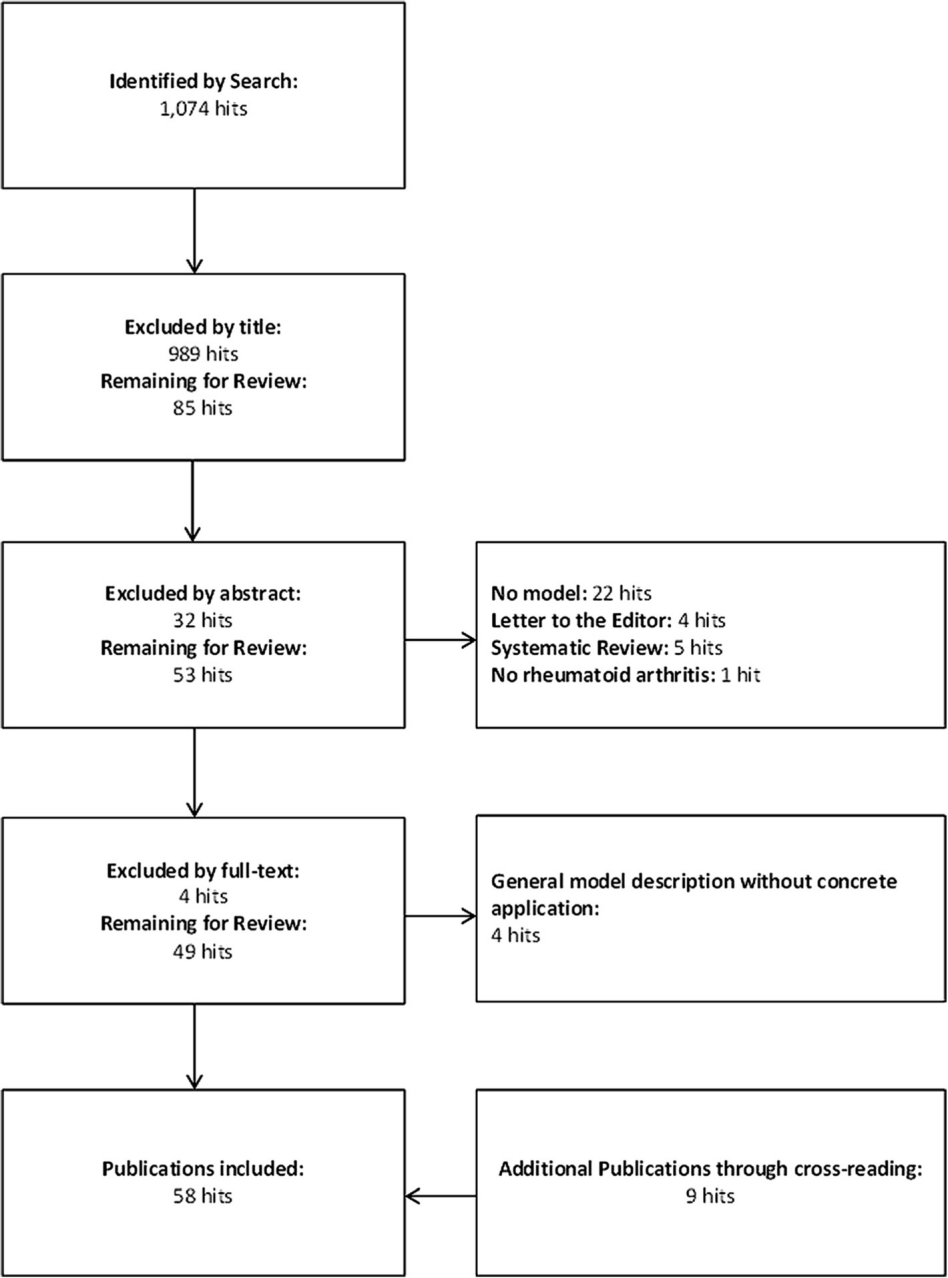
**Flow diagram of the conducted systematic review in PubMED.** Background colors represent the different modeling techniques (blue = decision trees, yellow = Markov models, orange = ISM, green = DES) and bold letters and bright colors indicate an independently developed model.

To identify developments for the use of modeling techniques in RA, a systematic literature search was conducted January 14, 2014 in PubMED via Medline using “Medical Subject Heading” (MeSH) terms. The search yielded 1,074 hits for (“Arthritis, Rheumatoid” [Mesh] AND “Economics” [Mesh]) with no filters or limitations applied. The review process is depicted in Figure 1. Publications were excluded if they were not using modeling techniques or not applied to specific treatment options. Publications were included if patients with RA were part of the study population.

Identified systematic reviews were used for cross validation of identified studies. The main focus of the review process was to identify analysis techniques applied in the modeling of the course of RA treatment. Firstly, the publications were categorized by modeling technique, country and year and screened for information on specific modeling parameters (e.g. cycle length). Secondly, clinical measures used to describe the course of the disease within the model were extracted and supplemented by the measurements used to reflect changes in those health states. Additionally, the sources of the efficacy data and the base-case results were extracted and are presented in Additional files 1 and 2.

### Rheumatoid arthritis

#### Epidemiology

Rheumatoid Arthritis (RA) is an inflammatory, auto-immune disease of chronic course with unknown etiology. The prevalence of RA is estimated to range between 0.5 to 1 percent in western populations whereby women are more often affected by the disease with a ratio of 3:1. Concerning the incidence, there is some evidence of a decreasing RA rate due to a later onset of the disease [3]. Nevertheless, increased mortality of RA patients in comparison to the general population remains unchanged and the gap may be even increasing [4].

#### Etiology of the disease

The clinical course of the disease usually starts with unspecific symptoms like increasing exhaustibility, anorexia and weight loss, followed by specific symptoms of painfully swollen joints. Usually, the affection of joints occurs in a symmetrical manner and mostly affects joints of the hands and knees. As movement, stretching and pressure aggravate pain, patients tend to take a relieving posture often resulting in stiffness of the affected joints [5]. The course of the disease is characterized by periodic phases of higher and lower disease activity, which can be measured via the Disease Activity Score (DAS) or the DAS with 28-joint count (DAS28) [6]. The disease state can be classified using the scoring system published by the American College of Rheumatology (ACR) [7] (see also newest version from Alehata et al. (2010) [8]). In the long run, the chronic inflammation leads to damaged joints and destruction of cartilage [9]. Thereby, the functional disability of RA patients increases on a diminishing scale and is often measured via the disability index of the Health Assessment Questionnaire (HAQ). The HAQ is one of the standard domain specific quality of life instruments in rheumatology that gives a detailed impression of specific functional impairments adding to the burden of disease [10,11]. A premature mortality of RA is primarily cause-specific, but may also be influenced by adverse events of anti-rheumatic drugs [12].

### Treatment options and treatment guidelines

In general, treatment options for RA include patient education, pharmacotherapy, physiotherapy and surgery [13]. Within pharmacotherapy, drugs can be distinguished into Disease-Modifying-Anti-Rheumatic-Drugs (DMARDs) and symptom relief medications such as corticosteroids. DMARDs are recommended as monotherapy for patients with early RA in remission or with low disease activity or as combinational therapy in patients with established RA [14]. Among the DMARDs, so-called biologics (e.g. anti-TNF compounds) play a major role today in the treatment algorithm of active RA patients. The American College of Rheumatology (ACR) treatment guidelines recommend different strategies for RA patients depending on the time since onset of disease (early vs. established RA), the grade of disease activity (low, moderate or high) and whether the patient is with or without poor prognosis [14]. Disease activity should be reassessed at regular intervals and drug treatment should be modified or switched in the case of adverse events, non-response or loss of efficacy over time. More or less the same approaches to treatment are recommended by guidelines of *The European League Against Rheumatism* (EULAR).

Treatment of RA undoubtedly has become more efficacious after the introduction of biologic treatment alternatives but it has also become a major area of concern to health care payers when it comes to the budget impact. In such scenarios it is of paramount importance to explore relative cost effectiveness of different alternatives or approaches to treatment to sort out viable options not only for health care payers, but ultimately also for patients who usually have to finance the health care system one way or the other.

### Health economic modeling techniques

Health economic models are common tools for decision analyses in health administrative bodies and are increasingly used in health economic research. Hereby, different model types might be distinguished from relative simple models such as decision trees to more advanced modeling exercises (e.g. agent-based models). Nowadays one of the most commonly used model type is that of a Markov-model.

#### Decision trees

The most simplistic model type used in health economics is the *decision tree*. Within a decision tree the course of a disease is displayed as a hierarchical, one-directional tree with either a chance node or a terminal node at the end of each branch. These models can either be constructed for single patients or cohorts, but without the possibility of interaction between individual subjects. Nevertheless, decision trees only allow for the analysis of fixed time frames, making the approach problematic in use for diseases with varying length in certain health states. Furthermore, to encounter heterogeneity between patient-groups, input parameters need to be changed and the model needs to be run separately for every subgroup.

#### Markov models

In health economic *Markov-chain modeling,* the course of a disease is described by (various) discrete health states. Over time, cohorts move through or between these health states at the end of a cycle of a fixed time interval. Transition probabilities determine the number of patients remaining in a health state at the end of a cycle and also the number of subjects moving to other connected potential health states. Markov models are usually run until all patients of a cohort reached an absorbing state (e.g. death) or maximum number of cycles. The number of patients over all health states needs to stay constant in all cycles and patients are only allowed to be in one health state during a cycle [15]. It is also important to note that there are no interaction or individual decisions modeled between the different subjects in a Markov model. As the disease is mimicked via the creation of a limited number of distinct health states, available data needs to be adjusted to represent the clinical course, costs and outcomes at those fixed health states. Similar to a decision tree Markov models can analyze subgroups only by adjustment of model parameters and repeated runs of the model to cover questions of heterogeneity. Although it is possible to cover small numbers of subgroups of to address questions of heterogeneity by additional health states, it is feasible to analyze high numbers of subgroups by repeated runs of the model with different parameter sets for technical boundaries.

#### Individual Sampling Methods (ISM)

Another method to address problems of heterogeneity is to use individual sampling models [1]. In accordance to Markov models, individual sampling models or Monte Carlo simulations consist of different distinct states, but as only single patients are sent through the model, attributes of patients may be changed over time thus changing transition probabilities or other input parameters. This way individual sampling models overcome the need for a disproportional number of health states to track changes over time. For following references in this paper with respect to this method the main distinction of the individual sampling method and DES methods is defined as the use of fixed cycle lengths.

#### Discrete Event Simulations (DES)

In contrast to the above described modeling techniques, *discrete event simulation* (DES) focuses on the treatment pathway of a patient rather than modeling the course of the disease itself. In short, single entities (e.g. patients, health care professionals) with certain attributes (e.g. age, gender, disease severity) experience certain events (e.g. disease progression, hospital admission) based on their attributes, and, as a result of that, experience benefits or harms and consume resources. A strong emphasis of DES lies on the modeling of scarce resources and the behavior of entities competing or waiting for the availability of those resources [16]. In this context, an interaction between entities, e.g. patients, is possible as they can for example form waiting queues and access resources following pre-defined rules. Also, a main advantage of DES is its capability of the inclusion of patient characteristics, thus accounting for heterogeneity [16].

Even though DES is one of the more advanced methods used in health economic evaluation it still has some disadvantages attached. Firstly, DES is rather process-oriented leaving out interactions between entities which are not connected to resources. Secondly, it leaves the study subjects in a rather passive role making them flow through the system instead of letting them decide independently [17]. Additionally, the higher complexity of the model comes with a much higher demand on input data, which in many situation will not be easily accessible (e.g. patient-level data from clinical trials). Also, the sampling from distributions for each parameter for a large number of individuals involves model calibration, which is still under methodological development.

### ISPOR Guidelines on good modeling practice (health transition modeling and DES-modeling)

Besides the decision tree method, the International Society for Pharmacoeconomics and Outcomes Research (ISPOR) provides guidelines for good modeling practice for each of the above mentioned modeling techniques. Concerning the choice of the model, the authors recommend a Markov cohort simulation, if the number of necessary health states which depict the health problem remains manageable. If some aspect of the health problem cannot be reproduced in an appropriate way, an ISM modeling exercise should be chosen, but sequential decisions on treatment options should not be considered in the same model [18]. DES is recommended as first choice to model problems caused by constrained or limited resources. In addition, DES is seen as being also suitable to model time to one or multiple events stochastically and considering many characteristics of a patient influencing time. Thereby, the trade-off between the detailing of the model and the availability of data should be described and changes to the model structure or the influence of expert opinions filling data gaps should be explored and reported [19].

## Review

### Cost-effectiveness modeling in RA

#### Summary over all identified analyses

**Figure 2:**
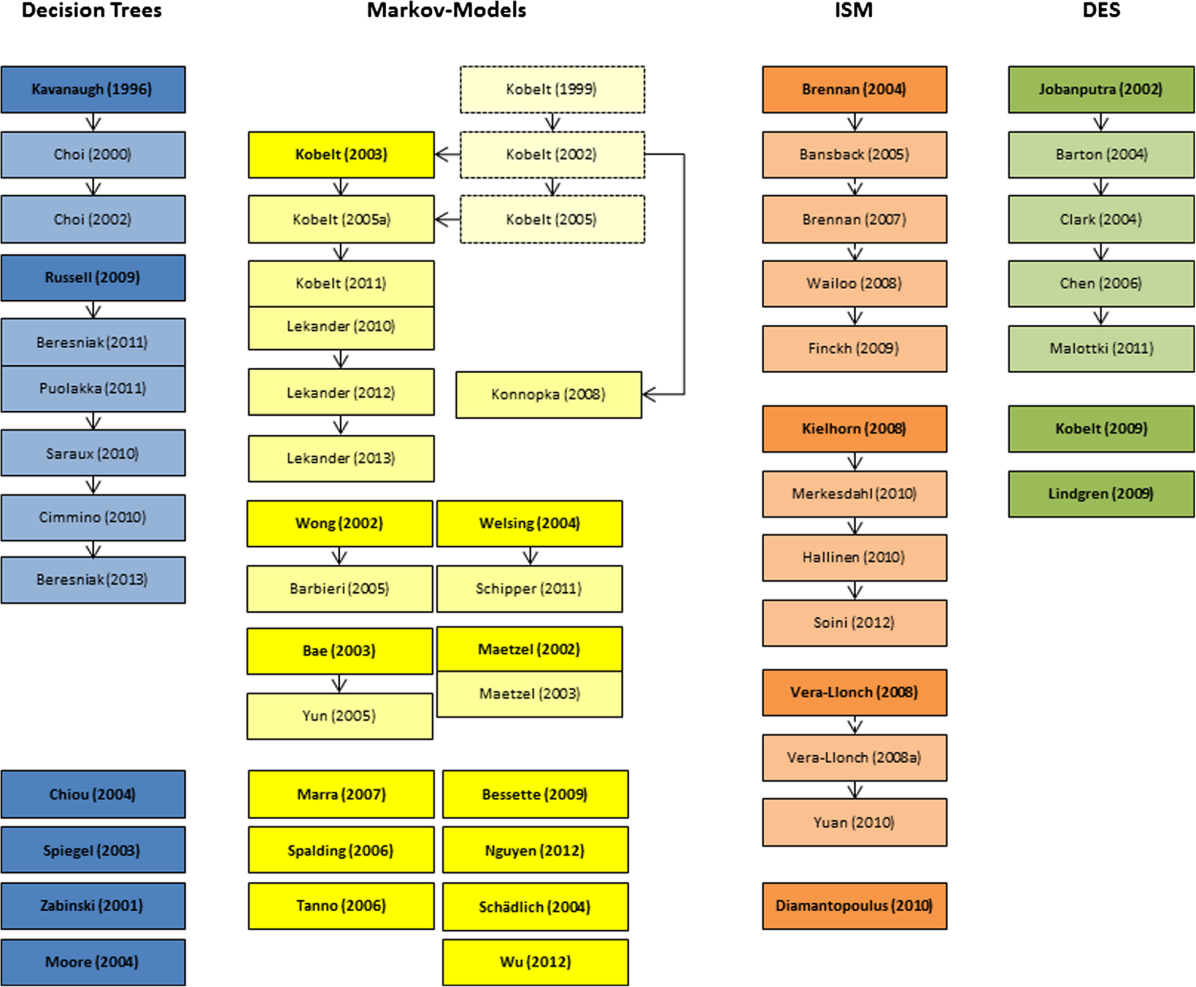
**Family tree of analyzed publications.** Background colors represent the different modeling techniques (blue = decision trees, yellow = Markov models, orange = ISM, green = DES) and bold letters and bright colors indicate an independently developed model.

Via the literature search n = 58 publications between 1996 and 2012 could be identified. 13 studies used a decision tree approach, 25 studies Markov-modeling, 13 studies an individual sampling method (ISM) and the DES-technique was applied seven times. As many of those studies are adaptations or enhancements of existing models, a family tree of all publications is displayed in Figure 2, where publications in bold letters represent the original model within a tree of references.

With 12 out of 25 studies, the highest number of independently developed models can be found for Markov-models with a similar share of six out of 13 for decision tree-studies. In contrast, four out of 13 ISMs and three out of seven DES-studies might be considered independently developed models.

On average the decision tree models have the shortest time horizons of all model types, besides Moore et al., Spiegel et al. and Zabinski et al. apply a life-time horizon [20-22]. The Markov models range from one year to life-time mostly applying a six month cycle length (three to twelve months), whereas the DES and ISM models, with one exception, take a life-time perspective. A summary of study characteristics can be found in Table 1 (sorted by model type).

**Table 1:** **Summary of study characteristics**

**Publication**	**Model type**	**Perspective**	**Cycle length**	**Time horizon**	**DAS**	**PSA**	**Efficacy measure**	**Clinical measure**
**Beresniak 2011** [26] Spain	Decision Tree	TTP	6 months	2 years	No	Yes	Effect on DAS28-Score	DAS28-Score
**Beresniak 2013** [30] Germany	Decision Tree	TTP	6 months	2 years	No	No	Effect on DAS28-Score	DAS28-Score
**Chiou 2004** [31] USA	Decision Tree	TTP	6 months	1 year	Yes	No	ACR	ACR/VAS-Score
**Choi 2000** [24] USA	Decision Tree	Societal	Not applicable	6 months	Yes	No	ACR	-
**Choi 2002** [16] USA	Decision Tree	Societal	Not applicable	6 months	Yes	No	ACR	ACR Response/ No Response
**Cimmino 2011** [27] Italy	Decision Tree	TTP	6 months	2 years	No	Yes	Effect on DAS28-Score	DAS28-Score
**Kavanaugh 1996** [23] USA	Decision Tree	Not stated (Societal)	Not applicable	6 months	Yes	No	-	Response, Partial Response, No Response
**Moore 2004** [20] UK	Decision Tree	TTP	Not applicable	life-time	Yes	Yes	Reduction in Complications	Complications
**Puolakka 2012** [28] Finnland	Decision Tree	TTP	6 months	2 years	No	Yes	Effect on DAS28-Score	DAS28-Score
**Russell 2009** [25] Canada	Decision Tree	TTP	?6 months	2 years	No	Yes	Effect on DAS-Score	DAS-Score
**Saraux 2010** [29] France	Decision Tree	TTP	6 months	2 years	No	Yes	Effect on DAS28-Score	DAS28-Score
**Spiegel 2003** [21] USA	Decision Tree	TTP	Not applicable	life-time	Yes	Yes	Reduction in Complications	Complications
**Zabinski 2001** [22] Canada	Decision Tree	TTP	Not applicable	life-time	Yes	No	Reduction in Complications	Complications
**Bae 2003** [47] Korea	Markov Model	Not stated	12 months	life-time	Yes	No	Reduction in Complications	Complications
**Barbieri 2005** [45] UK	Markov Model	TTP	6 months	life-time	Yes	No	Effect on HAQ-Score	HAQ-Score
**Bessette 2009** [55] Canada	Markov Model	TTP	1 month	5 years	Yes	No	Reduction in Complications	Complications
**Kobelt 1999** [33] Sweden	Markov Model	Not stated (Societal)	12 months	5 years	No	No	Effect on HAQ-Score	HAQ-Score
**Kobelt 2002** [34] Sweden & UK	Markov Model	Societal	12 months	10 years	No	No	Effect on HAQ-Score	HAQ-Score
**Kobelt 2003** [32] Sweden & UK	Markov Model	Not stated (Societal)	12 months	10 years	Yes	No	Effect on HAQ-Score	HAQ-Score
**Kobelt 2005** [35] Sweden	Markov Model	not stated	not stated	10 years	No	Yes	not stated	HAQ Score & VAS Score
**Kobelt 2005a** [36] Sweden	Markov Model	Societal	12 months	10 years	Yes	Yes	Effect on HAQ-Score	HAQ-Score
**Kobelt 2011** [37] Sweden	Markov Model	Societal	6 months	10 years	No	Yes	Effect on HAQ- & DAS28 Score	HAQ-Score & DAS28-Score
**Lekander 2010** [39] Sweden	Markov Model	Societal	?12 months	20 years	Yes	No	Effect on HAQ- & DAS28 Score	HAQ-Score & DAS28-Score
**Lekander 2012** [38] Sweden	Markov Model	Societal	12 months	20 years	Yes	Yes	Effect on HAQ- & DAS28 Score	HAQ-Score & DAS28-Score
**Lekander 2013** [40] Sweden	Markov Model	Not stated	12 months	10 years	No	Yes	Effect on HAQ- & DAS28 Score	HAQ-Score & DAS28-Score
**Maetzel 2002** [49] Canada	Markov Model	TTP	6 months	5 years	No	Yes	-	ACR20 Response
**Maetzel 2003** [48] Canada	Markov Model	TTP	3 months	5 years	Yes	No	Reduction in Complications	Complications
**Marra 2007** [52] Canada	Markov Model	Societal	1 week	10 years	No	Yes	Effect on HAQ-Score	HAQ-Score
**Nguyen 2012** [53] USA	Markov Model	TTP	3 months	5 years	Yes	Yes	-	ACR50 Response
**Schädlich 2004** [55] Germany	Markov Model	TTP	6 months	3 years	Yes	No	-	ACR20 Response
**Schipper 2011** [42] The Netherlands	Markov Model	Societal	3 months	5 years	Yes	Yes	Effect on DAS28-Score	DAS28-Score
**Spalding 2006** [50] USA	Markov Model	Societal	12 months	Life-time	Yes	No	Effect on HAQ-Score	HAQ-Score
**Tanno 2006** [51] Japan	Markov Model	Not stated (Societal)	6 months	Life-time	Yes	No	ACR	HAQ-Score
**Welsing 2004** [41] The Netherlands	Markov Model	not stated	3 months	5 years	Yes	Yes	EULAR &ACR	DAS-Score
**Wong 2002** [43] USA	Markov Model	Societal	6 months	Life-time	Yes	No	Effect on HAQ-Score	HAQ-Score
**Wu 2012** [56] China	Markov Model	TTP	6 months	Life-time	Yes	Yes	-	ACR20/50/70 Response
**Yun 2005** [47] Korea	Markov Model	Societal	12 months	Life-time	Yes	No	Reduction in Complications	Complications
**Bansback 2005** [57] Sweden	ISM	Not stated (TTP)	6 months	Life-time	Yes	Yes	ACR	HAQ-Score
**Brennan 2004** [15] UK	ISM	Societal	6 months	Life-time	Yes	No	ACR	HAQ-Score
**Brennan 2007** [58] UK	ISM	TTP	6 months	Life-time	Yes	Yes	EULAR	HAQ Score
**Diamantpoulus 2012** [68] Italy	ISM	TTP	6 months	Life-time	Yes	Yes	-	ACR20/50/70 Response
**Finckh 2009** [60] USA	ISM	Societal	6 months	Life-time	Yes	Yes	Effect on HAQ-Score	HAQ-Score
**Hallinen 2010** [63] Finnland	ISM	Societal	6 months	100 years	Yes	Yes	Effect on HAQ-Score	HAQ-Score
**Kielhorn 2008** [61] UK	ISM	TTP	6 months	Life-time	Yes	Yes	ACR	HAQ Score
**Merkesdahl 2010** [62] Germany	ISM	TTP	6 months	100 years	Yes	Yes	ACR	HAQ-Score
**Soini 2012** [64] Finnland	ISM	Societal	6 months	Life-time	Yes	Yes	ACR	HAQ Score
**Vera-Llonch 2008** [65] USA	ISM	TTP	3 months	Life-time	Yes	Yes	Effect on HAQ-Score	HAQ-Score
**Vera-Llonch 2008a** [66] USA	ISM	Not stated	3 months	Life-time	Yes	Yes	Effect on HAQ-Score	HAQ-Score
**Wailoo 2008** [59] USA	ISM	TTP	6 months	Life-time	Yes	Yes	ACR	HAQ-Score
**Yuan 2010** [67] USA	ISM	TTP	3 months	Life-time	Yes	Yes	Effect on HAQ-Score	HAQ-Score
**Barton 2004** [70] UK	DES	Not stated	Not applicable	Life-time	No	No	Effect on HAQ-Score	HAQ-Score
**Chen 2006** [71] UK	DES	TTP	Not applicable	Life-time	Yes	No	Effect on HAQ-Score	HAQ-Score
**Clark 2004** [17] UK	DES	TTP	Not applicable	Not stated	Yes	No	Effect on HAQ-Score	HAQ-Score
**Jobanputra 2002** [69] UK	DES	Not stated	Not applicable	Life-time	Yes	No	Effect on HAQ-Score	HAQ-Score
**Kobelt 2009** [74] Sweden	DES	Societal	Not applicable	10 years	Yes	Yes	Effect on HAQ- & DAS28 Score	HAQ-Score & DAS28-Score
**Lindgren 2009** [73] Sweden	DES	Societal	Not applicable	Life-time	Yes	Yes	ACR	HAQ-Score & DAS28-Score
**Malottki 2011** [72] UK	DES	Not stated (TTP)	Not applicable	Life-time	Yes	Yes	Effect on HAQ-Score	HAQ-Score

As can be seen in Table 2 decision tree models tend to compare treatment strategies involving etanercept, abatacept, adalimumab and methotrexate. Markov models focus on methotrexate, an unspecified TNF-biologic or gold. Individual sampling models mostly involve methotrexate and rituximab/methotrexate. Compared to the overall number of models, DES models are evaluating a much higher number of treatment strategies as more often treatment patterns or pathways are modeled. Eleven of the DMARD strategies as well as 14 TNF-alpha-antagonist strategies compared are not further specified.

**Table 2:** **Agents used in the model overall and differentiated by model type**

**Abbreviation**	**Full name**	**Overall**	**Decision trees**	**Markov models**	**ISM**	**DES**
**ABA**	Abatacept	13	12	0	1	0
**ABA/MTX**	Abatacept + Methotrexate	11	0	0	10	1
**ADA**	Adalimumab	29	20	3	2	4
**ADA/MTX**	Adalimumab + Methotrexate	20	1	2	13	4
**ANA**	Anakinra	1	1	0	0	0
**ANA/MTX**	Anakinra + Methotrexte	9	1	0	0	8
**AZA**	Azathioprine	48	0	8	0	40
**BSC**	Best Supportive Care	47	0	1	18	28
**BSC/MTX**	Best Supportive Care + Methotrexate	4	0	0	4	0
**BUC**	Bucillamine	2	0	2	0	0
**CEL**	Celecoxib	3	0	3	0	0
**CER/MTX**	Certolizumab + Methotrexate	1	0	1	0	0
**COR**	Corticosteroid	1	0	1	0	0
**COX-2**	selective COX-2 inhibitor	2	0	2	0	0
**CYC**	Ciclosporin	60	0	9	11	40
**CYC/MTX**	Ciclosporin + Methotrexate	35	1	0	0	34
**DIC**	Diclofenac	1	0	1	0	0
**DMARD**	any Disease-Modifying-Anti-Rheumatic-Drug	11	3	3	5	0
**ETA**	Etanercept	48	26	7	3	12
**ETA/MTX**	Etanercept + Methotrexate	18	2	3	9	4
**ETO**	Etoricoxib	1	1	0	0	0
**GOL/MTX**	Golimumab + Methotrexate	1	0	1	0	0
**Gold**		66	1	17	8	40
**HCQ**	Hydroxychloroquine	12	0	0	0	12
**HCQ/MTX**	Hydroxychloroquine + Methotrexate	20	1	8	0	11
**IBU**	Ibuprofen	1	0	1	0	0
**INF**	Infliximab	19	8	4	4	3
**INF/MTX**	Infliximab + Methotrexate	36	1	8	11	16
**LEF**	Leflunomide	66	1	16	9	40
**LEF/MTX**	Leflunomide + Methotrexate	1	0	1	0	0
**MTX**	Methotrexate	115	10	41	30	34
**NAP**	Naproxen	2	1	1	0	0
**NSAID**	(nonselective) nonsteroidal anti-inflammatory drugs	5	1	4	0	0
**NSAID/H2RA**	NSAID + H2 receptor antagonist	1	1	0	0	0
**NSAID/MIS**	NSAID + Misoprostol	2	1	1	0	0
**NSAID/PPI**	NSAID + proton pump inhibitor	3	1	2	0	0
**PEN**	Penicillamine	22	0	0	0	22
**PRD/MTX**	Prednisone/Prednisolone	0	0	0	0	0
**ROF**	Rofecoxib	1	0	1	0	0
**RTX**	Rituximab	14	7	6	0	1
**RTX/MTX**	Rituximab + Methotrexate	21	0	0	20	1
**SUL**	Sulfasalazine	48	2	10	2	34
**SUL/HCQ/MTX**	Sulfasalazine + Hydroxychloroquine + Methotrexate	14	1	2	0	11
**SUL/MTX**	Sulfasalazine + Hydroxychloroquine	20	0	4	0	16
**TNF**	TNF-inhibitor	14	1	9	2	2
**TOC/MTX**	Tocilizumab + Methotrexate	3	0	0	3	0
**OVERALL**		872	106	183	165	418

### Description of Models by Modeling Technique

#### Decision Trees

The earliest model included in this review is the decision tree analysis of Kavanaugh et al. [23], which has also been used by Choi et al. [24] and Choi et al. [16]. The decision tree splits up after choice of treatment. For each treatment, side-effects (minor and major) may occur. If no side-effects emerge a patient can either (partially or fully) respond or not respond following ACR criteria. For patients with minor side-effects it is also possible to respond to the treatment. The time-horizon is six months. Choi et al. [24] and Choi et al. [16] performed only deterministic sensitivity analyses, whereas Kavanaugh et al. [23] used Monte-Carlo simulation for one-way sensitivity analysis.

The second family of decision tree models was built by Russell et al. [25], Beresniak et al. [26], Cimmino et al. [27], Puolakka et al. [28], Saraux et al. [29] and Beresniak et al. [30]. The trees depict the treatment of patients within four six-month intervals starting with a TNF-alpha-antagonist. After each interval patients either show signs of remission, in which case they stick with the current treatment, or they change to the next intervention. In all publications the DAS28 score is used to define effectiveness using the low-disease activity state (DAS28 ≤ 3.2) as primary end-point describing cost-effectiveness. Besides Beresniak et al. [30], all models perform probabilistic sensitivity analysis and all six models report an ICER for costs per day in a low disease activity state with confidence intervals.

Chiou et al. [31] use an independent decision tree-model to evaluate the cost-effectiveness of several biologic response modifiers. Similar to the Russell-models, intervals of six months are used for a total time horizon of one year. After the first six months the effectiveness of an intervention is measured using ACR response criteria categorized into no ACR, ACR20, ACR50 and ACR70 responses. After the second interval it is possible that patients develop no, mild, moderate or severe complications. Only deterministic, one-way-sensitivity analysis is performed.

In the publication by Spiegel et al. [21] a decision tree is applied to the question whether rofecoxib and celecoxib are cost-effective compared to non-selective non-steroidal anti-inflammatory drugs (NSAIDs) in the treatment of chronic arthritis. Over a life-time time horizon, the number of gastrointestinal (GI) complications, i.e. dyspepsia, ulcer bleeding and ulcer perforation, is modeled for each of the alternatives. Hence, the model does not focus on the efficacy of the alternatives on RA, but on the reduction on adverse events. The cost-effectiveness measure is calculated as incremental costs per QALY. A meta-analysis is used to obtain parameters for clinical efficacy and PSA is performed.

In a similar study, Zabinski et al. [22] compared celecoxib alone vs. several NSAIDs in osteoarthritic (OA) and RA patients, also focusing on adverse events following these treatments. Thereby gastrointestinal events are supplemented by “anaemia with occult bleeding”. The analysis is carried out for a time-horizon of 6 months and calculates costs from a third-party payer-perspective, but no ICER.

The decision tree constructed by Moore et al. [20] compares etoricoxib with several NSAID-strategies. Again, the main health states comprise gastrointestinal adverse events (“GI problems”, “no GI problems”), but with a more complex depiction of the following treatment patterns. As in the two former studies, not only RA-patients, but also OA-patients were included in the population of the analysis. An ICER from the perspective of the NHS is reported including deterministic and probabilistic sensitivity analysis.

### Markov models

The most adopted Markov-model is from Kobelt et al. [32], which is based on two earlier works also from Kobelt et al. [33,34]. Those studies are not included in this review as only hypothetical treatments are evaluated to justify the general use of Markov models in RA in those two studies. The authors built a model using seven states corresponding to HAQ-scores also including death with a time horizon of 10 years and a cycle-length of one year. In difference, the following studies by Kobelt et al. [35] and Kobelt et al. [36] reduced the number of disease states by one. Further, in the paper from 2011, Kobelt et al. [37] use a cycle length of six months and Lekander et al. [38] extended the time-horizon to 20 years. All following studies subdivided each health state by a high or low VAS-Score or high or low disease-activity measured by the DAS28-score. Whereas Kobelt et al. [32] analyze the cost-effectiveness of infliximab, the model from Kobelt et al. [36] does not evaluate the cost-effectiveness of a specific drug, but constitutes an advancement compared to Kobelt et al. [34]. Kobelt et al. and Kobelt et al. investigate the cost-effectiveness of etanercept in combination with methotrexate and etanercept monotherapy. In addition, Lekander et al. extended this analysis by grouping TNF-alpha-inhibitors and Lekander et al. [40] analyzed the cost-effectiveness of infliximab. Another application of the model compared the results of the model for three different trial cohorts (Lekander et al. [39]). From Kobelt et al. on, probabilistic sensitivity analyses (PSA) has been used [39,40].

Welsing et al. [41] and Schipper et al. [42] are using the four Markov-states “remission”, “low disease activity”, “moderate disease activity” and “high disease activity” corresponding to DAS28-scores, although with different classifications. Patients may change the treatment after inadequate response. Both studies use a cycle length of three months and a time horizon of five years. PSA was performed using 1,000 iterations or 1,000 patients, respectively.

The original Markov-model from Wong et al. [43] has later been adapted by Barbieri et al. [44] for a longer time horizon. In both models, four different health states are defined using HAQ-scores for each of the five treatment options, thus resulting in 21 health states with the inclusion of death. The models extrapolate the results of the ATTRACT trial for infliximab from 54 weeks to a life-time horizon using a cycle length of six months. The first model has been validated in an earlier publication. [45] No PSA was performed.

Bae et al. [46] developed a first model for Korea to examine the cost-effectiveness of corticosteroids compared to NSAIDs and COX-2 inhibitors in RA patients. Over a life-time patients are moving through seven corticosteroid-specific or seven NSAID-specific complication-health states and the absorbing “death”-state in twelve month cycles. An ICER is given as costs per QALY and a deterministic sensitivity analysis was performed. The model has been adopted by Yun and Bae [47] to compare several NSAID and one COX-2 treatment strategy. Therefore, the health states concerning the NSAID-specific complications were kept in the model and supplemented by health states depicting treatment patterns.

In their first publication Maetzel et al. [48] focus on RA patients alone and the cost-effectiveness of adding leflunomide to a treatment sequence of DMARDs in Canada. The health states are divided into “ACR20-response” or “no ACR20-response” leading to continuation of current treatment and “severe AE” or “lack of efficacy” leading to the next treatment in the sequence. The result is given as an ICER (costs per QALY) and uncertainty is captured by probabilistic sensitivity analysis. In their second study, Maetzel et al. [49] analyze the cost-effectiveness of NSAIDs compared to COX-2 inhibitors. GI events are modeled for OA or RA patients over 12 health states, each supplemented by a decision tree reflecting the treatment pattern following the specific health state. The ICER is calculated as costs per QALY from a third-party payer perspective and only a deterministic sensitivity analysis is performed.

The model by Spalding et al. [50] includes three different Markov-states. Patients start with a specific first-line or initial drug therapy and move to a pooled treatment state or to death from either of the two other states. For each of the two treatment states certain costs and a specific reduction in the HAQ-score are assumed per 12 month cycle. Again, no PSA was conducted.

Tanno et al. [51] are presenting the only model for Japan. Similar to the model family around Kielhorn et al. [61], the Markov-states represent the treatment options and patients move in six months cycles to the next treatment in sequence, if they fail an ACR20 response. The authors include age- and sex-specific excess mortality due to RA as an exponential function of the HAQ-score. Patients are also assumed to have failed an initial therapy with bucillamine, whereas the main interest is in the cost-effectiveness of etanercept. Only one-way sensitivity analyses were performed and reported as tornado-plot.

The first intention of Marra et al. [52] lies in the cost-effectiveness of infliximab in combination with methotrexate compared to methotrexate monotherapy. Secondly, the authors also explore the influence of different quality of life questionnaires (HUI-2, HUI-3, SF-6D, EQ-5D) on health utility and therefore on cost-effectiveness. Within a time-horizon of 10 years patients move between 25 health states, as defined corresponding to HAQ-scores from zero to three in steps of 0.125 (similar to the BRAM approach described later) plus death in cycles of one week. PSA was performed and the differences between the different questionnaires are clearly demonstrated.

Nguyen et al. [53] present a similar but independently developed model to Spalding et al. [50]. In the model, patients start with the treatment of one of five different TNF-α antagonists in the first health state and either move to the ACR50-response health state or a non-response health state. Patients in the non-responder health state are assumed to switch to tocilizumab as second-line treatment. Patients can die from all three health states. The cycle length of the model is three months and the time horizon is five years. One-way deterministic as well as probabilistic sensitivity analyses were applied.

The study of Bessette et al. [54] compares celecoxib as 1^st^, 2^nd^ and 3^rd^ line treatment option in addition to different NSAID-strategies. In similarity to other studies with a comparable research question, the health states consider mainly GI events supplemented by one health state for cardiovascular AEs. In the case of an AE, patients continue to the next treatment option in the sequence. Of all Markov-models Bessette et al. are using the shortest cycle length with one month (time-horizon: five years).

The German publication from Schädlich et al. [55] reports the results from an adaption of the “The Avara® interactive model”, on which no further information or publication could be found. The cost-effectiveness of additional leflunomide in four different DMARD-treatment sequences is analyzed using ACR20-response to differentiate the health states. The model is run for six cycles of six months cycle length. The ICER for each sequence with leflunomide is calculated for the corresponding sequence without leflunomide.

Wu et al. [56] report the only cost-effectiveness model found for China comparing seven different treatment sequences. Health states are defined using ACR20/50/70 response criteria and the next treatment option in the particular sequence is selected after poor remission or adverse events. The results are presented as costs per QALY in a cost-effectiveness frontier. Deterministic as well as probabilistic sensitivity analyses are performed.

### Individual sampling models

Brennan et al. [15] developed the first individual sampling model. Thereby, certain events for patients of a certain age, sex and HAQ-Score are calculated using fixed time intervals of six months for DMARDs and three months for etanercept, respectively. In detail, patients who failed two conventional DMARDs are assumed to receive a certain treatment which might lead to a response according to the ACR20 criteria, thus continuing treatment until loss of efficacy or an adverse event, no response or death. The responders may continue the treatment and non-responders move on to the next treatment in sequence. Costs and utilities for each patient are documented during the modeling process. Bansback et al. [57] describe a slightly modified model for Sweden, where adverse events and the loss of efficacy are explicitly included in the first cycle in which patients receive a new treatment. Brennan et al. [58] use their model from 2004 with data from the British Society for Rheumatology Biologics Registry (BSRBR) for 8,000 patients and Wailoo et al. [59] with data from the National Database of Rheumatic Diseases (NDB) for 10,000 US-patients. The latter model has been modified by Finckh et al. [60] to compare three different treatment strategies including DMARDs (conventional and biologic) and other pharmaceutical and non-pharmaceutical interventions.

In the individual sampling-model by Kielhorn et al. [61] patients start after inadequate response to two biological DMARDs and begin with states related to a certain treatment sequence. For each treatment patients may enter five different health states (including death) defined by the ACR-Score and move to the next treatment if they show no response. If they completed all treatment options patients move to a palliative care state. The time-horizon is 100 years with a cycle-length of 6 months. Merkesdal et al. [62], Hallinen et al. [63] and Soini et al. [64] are using the same model structure for country specific analyses. The analyses were conducted using 10,000 patients and 3,000 patients in both Finnish studies, respectively. Additionally, PSA was performed using 1,000 iterations in all models.

Vera-Llonch et al. [65] and Vera-Llonch et al. [66] lean their individual sampling models against Brennan et al. [58]. Both studies are evaluating the cost-effectiveness of abatacept, using data from the clinical AIM or ATTAIN trials with methotrexate or an unspecified DMARD as comparator, respectively. Patients in the model who are assumed to start treatment with abatacept are considered to continue on treatment after 6 months of therapy if they show a HAQ-score improvement of at least 0.50, no side-effects (no cost and utility affects assumed), no co-morbid conditions and no surgery. The model analyses costs and QALYs of 1,000 female patients between 55 and 64 years over ten years and life-time with a cycle length of three months. Changes on HAQ-scores were considered as multiples of 0.125. The models were adopted by Yuan et al. [67] to analyze the cost-effectiveness of abatacept and rituximab.

The only non-adopted ISM was done by Diamantopoulus et al. [68]. The model was developed to evaluate the cost-effectiveness of tocilizumab in addition to a sequence of DMARDs and TNF-inhibitors from an Italian third-party payer perspective. 10,000 patients are followed over a life-time. Treatment efficacy is measured via the ACR20/50/70 response criteria and patients continue to the next treatment option after no response. ICERs are calculated for all sequences and deterministic as well as probabilistic sensitivity analyzes are performed.

### Discrete event simulations

Most DES models are developed for the United Kingdom and used within the Health Technology Assessment process. The first DES model has been developed by Jobanputra et al. [69] to assess the cost-effectiveness of infliximab and etanercept and is known as the Birmingham Preliminary Model (BPM). Within the model, the time of an individual patient on a certain DMARD in a sequence of DMARDs is calculated and all corresponding costs and health outcomes are documented. Afterwards, depending on the remaining lifetime of a patient, the patient moves to the next treatment option or dies. For some treatments effects of certain toxicities are included in the model which may lead to changes in the treatment cascade.

Barton et al. [70] extended the model by allowing patients to stop the current treatment not only by continuing with the next DMARD in sequence or death, but also by undergoing a joint replacement and/or an increase in the HAQ-Score. Thereby, in order to decide which event occurs and ends the treatment, the time of each event is calculated separately and the event with the shortest timeframe is taken into account. Changes in the HAQ-score are modeled with an interval of 0.125.

Clark et al. [17] are using the *Birmingham Rheumatoid Arthritis Model* (BRAM) from Barton et al. to evaluate the cost-effectiveness of anakinra. Chen et al. [71] also use the BRAM for adalimumab, etanercept and infliximab, although joint replacements are left out of both latter models. The later adaption by Chen also includes individual improvements on HAQ-scores and early withdrawal from a treatment by patients is considered. The last adaptation of the BRAM is used by Malottki et al. [72] which made further improvements to the model by allowing for a non-linear translation of HAQ-scores to utility values and enabling probabilistic sensitivity analysis.

With the work from Lindgren et al. [73] only one non-UK DES-model could be identified. The authors analyze the cost-effectiveness of rituximab as second biologic agent in different treatment sequences. As with the BPM and BRAM, patients may be on or off treatment or dead, although the evaluated treatment sequences only include TNF-alpha-antagonists thus being shorter than in other DES models. When patients are on treatment they are either in a high or low DAS28 state and change to other states by treatment discontinuation, re-initiation, changing disease activity or death. The model was filled with data from the REFLEX trial and the SSATG-database. The model has been adopted by Kobelt et al. [74] with no major changes to analyze costs and outcomes for one sequence.

## Discussion

The present review analyzed 58 studies reporting cost-effectiveness modeling exercises on RA. The number of models using decision trees, Markov or ISM methods does not vary significantly, with fewer models using DES, but no trends in time could be observed with respect to a preferential use of certain model-types. Nevertheless, a higher number of Markov models are reported as independently conducted CEAs, whereas decision trees, ISM and DES tended to be adopted more often. This might be due to the increasing complexity and the higher computational requirements of ISM and DES when compared to the relatively wide-spread use of Markov models in health economic research.

Besides Lindgren et al. [73] and Kobelt et al. [74], all DES studies primarily have been developed for the UK and were published as part of a health technology assessment (HTA), in which also models might be reviewed that are submitted by a pharmaceutical company to the National Institute for Clinical Excellence (NICE). The background given in the HTAs for the development of the Birmingham Preliminary Model (BPM) [64] and its updated versions – known as the Birmingham Rheumatoid Arthritis Model (BRAM) – is the ability of DES to more accurately depict comprehensive treatment sequences as they occur in RA patients (see Table 2) and a more flexible course of the disease. Barton et al. [70] also puts a strong emphasis on the timing on activities and how the BRAM deals with competing risks. [65] On the other hand an impending need for reliable data is stated, especially impact patterns on quality of life under certain treatments [64].

With regard to the differences in time and the different settings and perspectives, no valid direct comparisons between the results of the different modeling techniques could be performed and no publications could be identified performing a direct comparison between different methodologies with the same input data. However, two studies comparing DES and Markov models in HIV and depression could be found, respectively. Summarizing, Simpson et al. [75] report no considerable differences in the ICERs for a time-frame from five to ten years. However, as the DES model is capable to simulate a higher number of clinical parameters for HIV patients it seems to have a slightly better predictive ability than the Markov model, when the findings are compared to real life data. For depression, Le Lay et al. [76] compared a self-developed DES model with Markov models the authors identified in the literature. Though no ICERs are compared in the study, the authors also report the DES model to be able to incorporate more factors relevant to the treatment of depression than the Markov model, especially the patients’ history and their attitude towards different treatment options.

These examples illustrate the advantages DES models might also provide in their further application for RA. Under the consideration of their weaknesses, an even higher need (and mostly lack thereof) for reliable data and the resulting increased incorporation of expert opinion or even assumptions [2], a wider spectrum of outcome parameters might be presented to regulators as well as other decision makers. This could be a benefit but also a disadvantage, depending on the willingness of individual decision makers to look at even more complex data sets. Nevertheless, in any case it will be possible to reduce the wider variety of results from a DES to the information depth that usually comes with a Markov model.

However, the selection of the most appropriate model type, and therefore which simplification of reality is chosen, is a lively discussion. Even as there are guidelines on model selection (see for example [1,77]), following different guidelines may lead to different model types. Furthermore, questions on the relevance of certain parameters might be answered differently by modelers working on the same decision analytic problem, also leading to different approaches. Though simplicity is an advantage in models, if complex modeling techniques are available to tackle complex decision problems (e.g. life-time treatment sequences in RA) those model types should be applied [1]. In this context, the consideration of patient heterogeneity in economic evaluations [78] further encourages the use of more complex modeling techniques like DES [79].

Besides the models following Russell et al. [25], which use costs per day of low-disease activity score (LDAS), all models reviewed use a costs per QALY approach. The use of QALYs as their main outcome is justified with recommendations of regulatory bodies or with better comparability with other studies. In this context the burden of the disease might be modeled more comprehensively by the inclusion of necessary non-pharmaceutical treatments and socioeconomic consequences of the disease. In future studies also structural limitations in the health care provision for RA patients may be implemented in DES models. For example, the implications of a decreasing number of rheumatologists might be included in a DES framework using its ability to simulate queues given a maximal capacity a rheumatologist is able to take care of. This might also be relevant for time consuming and resource blocking therapies, such as intravenous infusions including monitoring for several hours or physical examinations.

## Conclusion

In conclusion, Markov models, ISM and DES are appropriate choices to model the cost-effectiveness of treatment options for RA patients, whereas decision trees are only able to produce results for short time horizons. Although ISM overcomes the lack of consideration of heterogeneity of RA patients in Markov (cohort) models, DES is able to produce even more detailed results. This might come at the cost of more assumptions needing to be incorporated in the model and decision makers should be aware of this. Therefore, extensive sensitivity analyses on input parameters as well as on structural components of the model and a clear declaration of data sources are necessary for a valid interpretation of the results.

The aim of this review was to provide an informative basis on the advantages and disadvantages of modeling techniques used to model RA and on the data sources for clinical parameters (see Additional files 1 and 2). If sufficient data are available, DES should be considered as the preferred modeling technique.

## Additional files

Additional file 1:
**Basecase-results of reviewed studies and treatment options compared.**
Additional file 2:
**Clinical Trial publications used for efficacy data in the CEA-models.**
