# Supplementary material for: Modeling rheumatoid arthritis using different techniques - a review of model construction and results
Source: Health Econ Rev. 2014 Sep 16;4:18. doi: 10.1186/s13561-014-0018-2 (PMC4502067; doi:10.1186/s13561-014-0018-2)
Supplement: Additional file 1: — Basecase-results of reviewed studies and treatment options compared. [file 13561_2014_18_MOESM1_ESM.docx]

### Basecase-results of reviewed studies and treatment options compared.

| **Author**  Primary outcome | **Year** | | **Treatment strategies compared** | **Results** | | | | |
| --- | --- | --- | --- | --- | --- | --- | --- | --- |
| **Bae** | **2003** | | NSAID |  |  |  |  | Comparator |
| per QALY |  | | COR |  |  |  |  | Dominated |
| **Bansback** | **2005** | |  |  |  |  | **ACR20** | **ACR50** |
| per QALY |  | | ADA/MTX |  |  |  | 40.875 € | 34.167 € |
|  |  | | ADA/MTX |  |  |  | 44.018 € | 34.922 € |
|  |  | | ETA/MTX |  |  |  | 51.976 € | 35.760 € |
|  |  | | INF/MTX |  |  |  | 64.935 € | 48.333 € |
|  |  | | ADA |  |  |  | 65.499 € | 51.561 € |
|  |  | | ETA |  |  |  | 42.480 € | 36.927 € |
|  |  | | DMARD |  |  |  | Comparator | Comparator |
| **Barbieri** | **2005** | | MTX |  |  |  |  | Comparator |
| per QALY |  | | INF/MTX |  |  |  |  | £23.936 |
| **Barton** | **2004** | | Sequence: (SUL - MTX -) Gold - AZA - PEN - HCQ - LEF - CYC - CYC/MTX - BSC |  |  |  | Comparator |  |
| per QALY |  | | Sequence: (SUL - MTX -) **ETA** - Gold - AZA - PEN - HCQ - LEF - CYC - CYC/MTX - BSC |  |  |  | £90.226 | £50.678 |
|  |  | | Sequence: (SUL - MTX -) **INF** - Gold - AZA - PEN - HCQ - LEF - CYC - CYC/MTX - BSC |  |  |  | £120.882 | Comparator |
| **Beresniak** | **2011** | | Sequence: ETA - **ABA** - ADA |  |  |  |  | Comparator |
| per day in LDAS |  | | Sequence: ETA - **RTX** - ADA |  |  |  |  | 91 € |
| **Beresniak** | **2013** | | Sequence: ADA - ABA - ETA |  |  |  |  | 633 € |
| per LDAS |  | | Sequence: ADA - RTX - ETA |  |  |  |  | 728 € |
|  |  | | Sequence: ADA - ETA - INF |  |  |  |  | 2.000 € |
|  |  | | Sequence: ADA - ETA - ABA |  |  |  |  | 1.067 € |
| **Bessette** | **2009** | | CEL - CEL/PPI |  |  |  | Comparator |  |
| per QALY |  | | NSAID - CEL - CEL/PPI |  |  |  | 80.077 € | Comparator |
|  |  | | NSAID - NSAID/PPI - CEL/PPI |  |  |  |  | Dominated |
| **Brennan** | **2004** | | Sequence: (MTX - SUL -) Gold - LEF - CYC |  |  |  |  | Comparator |
| per QALY |  | | Sequence: (MTX - SUL -) **ETA** - Gold - LEF - CYC |  |  |  |  | £1.633 |
| **Brennan** | **2007** | | DMARD |  |  |  |  | Comparator |
| per QALY |  | | TNF |  |  |  |  | £23.882 |
| **Chen** | **2006** | |  |  |  |  |  |  |
| per QALY | **Base** | | Sequence: MTX - SUL - SUL/MTX - SUL/HCQ/MTX - LEF - Gold - AZA - CYC - CYC/MTX - PEN - BSC | Comparator |  |  |  | Comparator |
|  | **TNF start** | | Sequence: **ADA** - MTX - SUL - SUL/MTX - SUL/HCQ/MTX - LEF - Gold - AZA - CYC - CYC/MTX - PEN - BSC | £52.600 | Comparator |  |  |  |
|  |  | | Sequence: **ETA** - MTX - SUL - SUL/MTX - SUL/HCQ/MTX - LEF - Gold - AZA - CYC - CYC/MTX - PEN - BSC | £49.400 | £43.100 |  | Comparator |  |
|  |  | | Sequence: **ADA/MTX**- SUL - SUL/MTX - SUL/HCQ/MTX - LEF - Gold - AZA - CYC - CYC/MTX - PEN - BSC | £171.000 | ADA alone more effective | Comparator |  | £3.830 |
|  |  | | Sequence: **ETA/MTX** - SUL - SUL/MTX - SUL/HCQ/MTX - LEF - Gold - AZA - CYC - CYC/MTX - PEN - BSC | £78.100 |  | £34.100 | Dominated | £26.200 |
|  |  | | Sequence: **INF/MTX** - SUL - SUL/MTX - SUL/HCQ/MTX - LEF - Gold - AZA - CYC - CYC/MTX - PEN - BSC | £654.000 |  |  |  | Comparator |
|  | **TNF third** | | Sequence: MTX - SUL - SUL/MTX - **ADA** - LEF - Gold - AZA - CYC - CYC/MTX - PEN - BSC | £141.000 | Comparator |  |  |  |
|  |  | | Sequence: MTX - SUL - SUL/MTX - **ETA** - LEF - Gold - AZA - CYC - CYC/MTX - PEN - BSC | £47.400 | £18.300 |  | Comparison inconclusive |  |
|  |  | | Sequence: MTX - SUL - SUL/MTX - **ADA/MTX** - LEF - Gold - AZA - CYC - CYC/MTX - PEN - BSC | £64.400 | £1.940 | Comparator |  | £2.560 |
|  |  | | Sequence: MTX - SUL - SUL/MTX - **ETA/MTX** - LEF - Gold - AZA - CYC - CYC/MTX - PEN - BSC | £49.800 |  | £31.500 | Comparator | £19.800 |
|  |  | | Sequence: MTX - SUL - SUL/MTX - **INF/MTX** - LEF - Gold - AZA - CYC - CYC/MTX - PEN - BSC | £139.000 |  |  |  | Comparator |
|  | **TNF last** | | Sequence: MTX - SUL - SUL/MTX - SUL/HCQ/MTX - LEF - Gold - AZA - CYC - CYC/MTX - PEN - **ADA** - BSC | £40.100 | Comparator |  |  |  |
|  |  | | Sequence: MTX - SUL - SUL/MTX - SUL/HCQ/MTX - LEF - Gold - AZA - CYC - CYC/MTX - PEN - **ETA** - BSC | £23.700 | £11.600 |  | Comparator |  |
|  |  | | Sequence: MTX - SUL - SUL/MTX - SUL/HCQ/MTX - LEF - Gold - AZA - CYC - CYC/MTX - PEN - **ADA/MTX** - BSC | £29.700 | ADA/MTX more effective/cost differ not sig. | Comparator |  |  |
|  |  | | Sequence: MTX - SUL - SUL/MTX - SUL/HCQ/MTX - LEF - Gold - AZA - CYC - CYC/MTX - PEN - **ETA/MTX** - BSC | £23.800 |  | £15.800 | Comparison inconclusive | £12.100 |
|  |  | | Sequence: MTX - SUL - SUL/MTX - SUL/HCQ/MTX - LEF - Gold - AZA - CYC - CYC/MTX - PEN - **INF/MTX** - BSC | £37.900 |  | ADA/MTX more effective/cost differ not sig. |  | Comparator |
| **Chiou** | **2004** | | ANA |  |  |  | Comparator |  |
| per QALY |  | | ETA |  |  |  | $13.387 |  |
|  |  | | ADA |  |  |  | Dominated |  |
|  |  | | ANA/MTX |  |  |  |  | Comparator |
|  |  | | ETA/MTX |  |  |  |  | $7.925 |
|  |  | | ADA/MTX |  |  |  |  | Dominated |
|  |  | | INF/MTX |  |  |  |  | Dominated |
| **Choi** | **2000** | |  |  | **ACR20** |  | **ACR70WR** |  |
| per QALY |  | |  |  | **MTX resistant** | **MTX naive** | **MTX resistant** | **MTX naive** |
|  |  | | no 2nd line agent |  | Comparator | Comparator | Comparator | Comparator |
|  |  | | ETA/MTX |  | $42.600 |  | $34.800 |  |
|  |  | | ETA |  | extended Dominated |  | extended Dominated |  |
|  |  | | CYC/MTX |  | Dominated |  | Dominated |  |
|  |  | | SUL/HCQ/MTX |  | $1.500 |  | $3.100 |  |
|  |  | | MTX |  | Dominated | $1.100 | Dominated | $1.500 |
| **Choi** | **2002** | |  |  |  |  | **ACR20** | **ACR70WR** |
| per QALY |  | | no 2nd line agent |  |  |  | Comparator | Comparator |
|  |  | | SUL |  |  |  | $11.500 | Dominated |
|  |  | | MTX |  |  |  | Dominant | Dominant |
|  |  | | LEF |  |  |  | Dominated | Dominated |
|  |  | | ETA |  |  |  | $41.900 | $40.800 |
| **Cimmino** | **2011** | | Sequence: ETA - **ABA** - ADA |  |  |  |  | 376 € |
| per LDAS |  | | Sequence: ETA - **RTX** - ADA |  |  |  |  | 456 € |
|  |  | | Sequence: ETA - ADA - **ABA** |  |  |  |  | 642 € |
|  |  | | Sequence: ETA - ADA - **INF** |  |  |  |  | 1.164 € |
| **Clark** | **2004** | | Sequence: SUL - MTX- LEF - ETA - INF/MTX - Gold - AZA - CYC - CYC/MTX |  |  |  |  | Comparator |
| per QALY |  | | Sequence: SUL - MTX- LEF - ETA - INF/MTX - **ANA/MTX** - Gold - AZA - CYC - CYC/MTX |  |  |  |  | £604.000 |
|  |  | | Sequence: SUL - MTX- LEF - ETA - INF/MTX - Gold - AZA - CYC - CYC/MTX - **ANA/MTX** |  |  |  |  | £131.000 |
|  |  | | Sequence: SUL - MTX- LEF - INF/MTX - Gold - AZA - CYC - CYC/MTX |  |  |  |  | Comparator |
|  |  | | Sequence: SUL - MTX- LEF - INF/MTX - **ANA/MTX** - Gold - AZA - CYC - CYC/MTX |  |  |  |  | £379.000 |
|  |  | | Sequence: SUL - MTX- LEF - INF/MTX - Gold - AZA - CYC - CYC/MTX - **ANA/MTX** |  |  |  |  | £105.000 |
|  |  | | Sequence: SUL - MTX - HCQ - Gold - LEF - ETA - INF/MTX - AZA - CYC - CYC/MTX |  |  |  |  | Comparator |
|  |  | | Sequence: SUL - MTX - HCQ - Gold - LEF - ETA - INF/MTX - **ANA/MTX** - AZA - CYC - CYC/MTX |  |  |  |  | £385.000 |
|  |  | | Sequence: SUL - MTX - HCQ - Gold - LEF - ETA - INF/MTX - AZA - CYC - CYC/MTX - **ANA/MTX** |  |  |  |  | £109.000 |
|  |  | | Sequence: SUL - MTX - HCQ - Gold - LEF - INF/MTX - AZA - CYC - CYC/MTX |  |  |  |  | Comparator |
|  |  | | Sequence: SUL - MTX - HCQ - Gold - LEF - INF/MTX - **ANA/MTX** - AZA - CYC - CYC/MTX |  |  |  |  | £278.000 |
|  |  | | Sequence: SUL - MTX - HCQ - Gold - LEF - INF/MTX - AZA - CYC - CYC/MTX - **ANA/MTX** |  |  |  |  | £106.000 |
| **Diamantopoulus** | **2012** | | Sequence: ADA/MTX - ETA/MTX - RTX/MTX - ABA/MTX - BSC |  |  |  |  | Dominant |
| per QALY |  | | Sequence: INF/MTX - ETA/MTX - RTX/MTX - ABA/MTX - BSC |  |  |  |  | 2.655 € |
|  |  | | Sequence: ETA/MTX - ADA/MTX - RTX/MTX - ABA/MTX - BSC |  |  |  |  | Comparator |
|  |  | | Sequence: **TOC/MTX** - ADA/MTX - RTX/MTX - ABA/MTX - BSC |  |  |  |  | Dominant |
|  |  | | Sequence: **TOC/MTX** - ETA/MTX - ADA/MTX - RTX/MTX - ABA/MTX - BSC |  |  |  |  | 17.119 € |
| **Finckh** | **2009** | | Pyramid Strategy |  |  |  | Comparator |  |
| per QALY |  | | Early DMARD |  |  |  | $4.849 | Comparator |
|  |  | | Early TNF |  |  |  | $727.894 | Dominated |
| **Hallinen** | **2010** | | BSC |  |  | Comparator |  |  |
| per QALY |  | | Sequence: RTX/MTX - BSC |  |  | 30.248 € | Comparator |  |
|  |  | | Sequence: **ADA/MTX** - BSC |  |  | 50.941 € |  |  |
|  |  | | Sequence: **ETA/MTX** - BSC |  |  | 50.372 € |  |  |
|  |  | | Sequence: **INF/MTX** - BSC |  |  | 36.121 € |  |  |
|  |  | | Sequence: **ABA/MTX** - BSC |  |  | 67.003 € |  |  |
|  |  | | Sequence: RTX/MTX - **ADA/MTX** - BSC |  |  | 38.235 € | 52.021 € |  |
|  |  | | Sequence: RTX/MTX - **ETA/MTX** - BSC |  |  | 38.938 € | 52.698 € |  |
|  |  | | Sequence: RTX/MTX - **INF/MTX** - BSC |  |  | 32.621 € | 37.013 € | Comparator |
|  |  | | Sequence: RTX/MTX - **ABA/MTX** - BSC |  |  | 46.367 € | 68.100 € |  |
|  |  | | Sequence: RTX/MTX - INF/MTX - **ADA/MTX** - BSC |  |  | 38.329 € |  | 54.701 € |
|  |  | | Sequence: RTX/MTX - INF/MTX - **ETA/MTX** - BSC |  |  | 38.785 € |  | 54.836 € |
|  |  | | Sequence: RTX/MTX - INF/MTX - **ABA/MTX** - BSC |  |  | 44.466 € |  | 70.616 € |
| **Jobanputra** | **2002** | | Sequence: SUL - MTX - Gold - AZA - PEN - HCQ - LEF - CYC - CYC/MTX - BSC |  |  |  | Comparator |  |
| per QALY |  | | Sequence: SUL - MTX -**ETA** - Gold - AZA - PEN - HCQ - LEF - CYC - CYC/MTX - BSC |  |  |  | £64.881 | £35.229 |
|  |  | | Sequence: SUL - MTX - **INF** - Gold - AZA - PEN - HCQ - LEF - CYC - CYC/MTX - BSC |  |  |  | £89.973 | Comparator |
| **Kavanaugh** | **1996** | | Gold |  |  |  |  | $6.725 |
| total costs |  | | MTX |  |  |  |  | $5.430 |
|  |  | | Hypothetical TNF |  |  |  |  | $9.411 |
| **Kielhorn** | **2008** | | Sequence: LEF - Gold - CYC - BSC/MTX |  |  |  |  | Comparator |
| per QALY |  | | Sequence: **RTX/MTX** - LEF - Gold - CYC - BSC/MTX |  |  |  |  | £14.690 |
|  |  | | Sequence: ADA/MTX - INF/MTX - LEF - Gold - CYC - BSC/MTX |  |  |  |  | Comparator |
|  |  | | Sequence: **RTX/MTX** - ADA/MTX - INF/MTX - LEF - Gold - CYC - BSC/MTX |  |  |  |  | £11.601 |
| **Kobelt** | **2003** | |  |  | **Sweden** |  | **UK** |  |
| per QALY |  | |  |  | **1 year** | **2 years** | **1 year** | **2 years** |
|  |  | | MTX |  | Comparator | Comparator | Comparator | Comparator |
|  |  | | INF/MTX |  | £3.440 | £16.100 | £34.800 | £48.200 |
| **Kobelt** | **2005a** | |  |  |  |  | **5 years** | **10 years** |
| per QALY |  | | MTX |  |  |  | Comparator | Comparator |
|  |  | | ETA |  |  |  | Dominated | Dominated |
|  |  | | ETA/MTX |  |  |  | 54.548 € | 37.331 € |
| **Kobelt** | **2011** | | MTX |  |  |  |  | Comparator |
| per QALY |  | | ETA/MTX |  |  |  |  | 13.500 € |
| **Konnopka** | **2008** | | Testing |  |  |  |  | 930 € |
| per QALY |  | |  |  |  |  |  |  |
| **Lekander** | **2010** | | BSC |  |  |  |  | Comparator |
| per QALY |  | | INF |  |  |  |  | 22.830 € |
| **Lekander** | **2012** | |  |  |  |  | **1st line** | **2nd line** |
| per QALY |  | | DMARD |  |  |  | Comparator | Comparator |
|  |  | | TNF combination |  |  |  | 54.066 € | 79.891 € |
|  |  | | TNF |  |  |  | 83.474 € | 120.091 € |
|  |  | | ETA combination |  |  |  | 49.879 € | 70.781 € |
|  |  | | ETA |  |  |  | 64.902 € | 84.151 € |
| **Lekander** | **2013** | |  |  |  | *Registry Cohort* | *Matched Cohort* | *RCT Cohort* |
| per QALY |  | | standard therapy |  |  | Comparator | Comparator | Comparator |
|  |  | | INF |  |  | 2.397 € | Cost Saving | 1.990 € |
| **Lindgren** | **2009** | | Sequence: TNF I - TNF II - TNF III |  |  |  |  | Comparator |
| per QALY |  | | Sequence: TNF I - RTX - TNF II - TNF II |  |  |  |  | Dominant |
| **Maetzel** | **2002** | | Sequence: MTX - MTX/SUL - MTX/SUL/HCQ - Gold - CYC |  |  |  |  | Comparator |
| per QALY |  | | Sequence: MTX - MTX/SUL - MTX/SUL/HCQ - **LEF** - Gold - CYC |  |  |  |  | $71.988 |
| **Maetzel** | **2003** | | ROF |  |  |  | $455.071 |  |
| per QALY |  | | NAP |  |  |  | Comparator |  |
|  |  | | CEL |  |  |  |  | Dominated |
|  |  | | IBU |  |  |  |  | Comparator |
|  |  | | DIC |  |  |  |  | $248.160 |
| **Malottki** | **2011** | | Sequence: LEF - Gold - CYC - AZA - BSC | Comparator |  |  |  |  |
| per QALY |  | | Sequence: **ADA/MTX** - LEF - Gold - CYC - AZA - BSC | £34.300 | Dominated | £46.400 | Dominated | £20.500 |
|  |  | | Sequence: **ETA/MTX** - LEF - Gold - CYC - AZA - BSC | £38.900 | Dominated | £37.800 | Comparator | £456.700 |
|  |  | | Sequence: **INF/MTX** - LEF - Gold - CYC - AZA - BSC | £36.100 | Dominated | £41.700 |  | Comparator |
|  |  | | Sequence: **RTX/MTX** - LEF - Gold - CYC - AZA - BSC | £21.100 | Comparator |  |  |  |
|  |  | | Sequence: **ABA/MTX** - LEF - Gold - CYC - AZA - BSC | £38.400 | £130.600 | Comparator |  |  |
| **Marra** | **2007** | | MTX |  |  |  |  | Comparator |
| per QALY | HUI-II | | INF/MTX |  |  |  |  | $53.429 |
|  | HUI-III | | INF/MTX |  |  |  |  | $32.018 |
|  | SF-6D | | INF/MTX |  |  |  |  | $69.829 |
|  | EQ-5D | | INF/MTX |  |  |  |  | $46.322 |
| **Merkesdahl** | **2010** | | Sequence: ADA/MTX - INF/MTX - Gold - CYC - MTX |  |  |  |  | Comparator |
| per QALY |  | | Sequence: **RTX/MTX** - ADA/MTX - INF/MTX - Gold - CYC - MTX |  |  |  |  | 24.517 € |
| **Moore** | **2004** | | ETO |  | Dominant | Dominant | £9.350 | £19.766 |
| per QALY |  | | NSAID |  |  |  |  | Comparator |
|  |  | | NSAID/H2RA |  |  |  | Comparator |  |
|  |  | | NSAID/PPI |  |  | Comparator |  |  |
|  |  | | NSAID/MIS |  | Comparator |  |  |  |
| **Nguyen** | **2012** | | ETA/MTX |  |  |  | Comparator |  |
| per QALY |  | | MTX |  |  |  | Dominated |  |
|  |  | | CER/MTX |  |  |  | $185.497 | Comparator |
|  |  | | ADA/MTX |  |  |  |  | Dominated |
|  |  | | GOL/MTX |  |  |  |  | Dominated |
| **Russell** | **2009** | |  |  |  |  | **Low disease activity** | **Remission** |
| per QALY |  | | Sequence: ABA - ETA - INF - DMARD |  |  |  | Dominant | Dominant |
|  |  | | Sequence: ETA - ABA - INF - DMARD |  |  |  | $12.514 | $16.829 |
|  |  | | Sequence: ETA - INF - ADA - DMARD |  |  |  | Comparator | Comparator |
| **Schaedlich** | **2004** | | Sequence: MTX - SSZ - Gold - AZA - HCQ |  |  |  |  | Comparator |
| per QALY |  | | Sequence: MTX - **LEF** - SSZ - Gold - AZA - HCQ |  |  |  |  | 5.027 € |
|  |  | | Sequence: SSZ - MTX - Gold - AZA - HCQ |  |  |  |  | Comparator |
|  |  | | Sequence: SSZ - **LEF** - MTX - Gold - AZA - HCQ |  |  |  |  | 55.445 € |
|  |  | | Sequence: HCQ - MTX - SSZ - Gold - AZA |  |  |  |  | Comparator |
|  |  | | Sequence: HCQ - **LEF** - MTX - SSZ - Gold - AZA |  |  |  |  | 67.270 € |
|  |  | | Sequence: HCQ - SSZ - MTX - Gold - AZA |  |  |  |  | Comparator |
|  |  | | Sequence: HCQ - **LEF** - SSZ - MTX - Gold - AZA |  |  |  |  | 31.752 € |
| **Schipper** | **2011** | | Sequence: **MTX** - **LEF** - TNF I - TNF II - RTX |  |  |  |  | Comparator |
| per QALY |  | | Sequence: **LEF/MTX** - **MTX** - TNF I - TNF II - RTX |  |  |  |  | 436.965 € |
|  |  | | Sequence: **MTX/TNF** **I** - TNF II - RTX |  |  |  |  | 114.982 € |
| **Soini** | **2012** | | Sequence: MTX - RTX/MTX - INF - LEF - CYC - MTX |  |  |  |  | Comparator |
| per QALY |  | | Sequence: **ETA/MTX** - RTX/MTX - INF - LEF - CYC - MTX |  |  |  |  | $21.257 |
|  |  | | Sequence: **TOC/MTX** - RTX/MTX - INF - LEF - CYC - MTX |  |  |  |  | $18.991 |
| **Spalding** | **2006** | | Sequence: **MTX** - Optimal Treatment |  |  |  |  | Comparator |
| per QALY |  | | Sequence: **ETA** - Optimal Treatment |  |  |  |  | $89.772 |
|  |  | | Sequence: **ADA** - Optimal Treatment |  |  |  |  | $63.769 |
|  |  | | Sequence: **ADA/MTX** - Optimal Treatment |  |  |  |  | $194.589 |
|  |  | | Sequence: **INF/MTX** - Optimal Treatment |  |  |  |  | $409.523 |
| **Spiegel** | **2003** | | NAP |  |  |  |  | Comparator |
| per QALY |  | | CEL/ROF |  |  |  |  | $275.809 |
| **Tanno** | **2006** | | Sequence: BUC - MTX - SUL - SUL/MTX - No DMARD |  |  |  |  | Comparator |
| per QALY |  | | Sequence: BUC - **ETA** - MTX - SUL - SUL/MTX - No DMARD |  |  |  |  | ¥ Mio. 2,50 |
| **Vera-Llonch** | | **2008** |  |  |  |  | **10 years** | **lifetime** |
| per QALY | |  | DMARD |  |  |  | Comparator | Comparator |
|  | |  | DMARD/ABA |  |  |  | $50.576 | $45.979 |
| **Vera-Llonch** | | **2008** |  |  |  |  | **10 years** | **lifetime** |
| per QALY | |  | Sequence: MTX |  |  |  | Comparator | Comparator |
|  | |  | Sequence: **ABA/MTX** - MTX |  |  |  | $47.910 | $43.041 |
| **Wailoo** | | **2008** | INF |  |  |  |  | Dominated |
| per QALY | |  | ETA |  |  |  |  | $92.058 |
|  | |  | ADA |  |  |  | $142.726 | Comparator |
|  | |  | ANA |  |  |  | Comparator |  |
| **Welsing** | | **2004** | Sequence: Usual Treatment |  |  | Comparator |  |  |
| per QALY | |  | Sequence: **LEF** - Usual Treatment |  |  | 10.584 € | Comparator |  |
|  | |  | Sequence: **TNF** - Usual Treatment |  |  |  |  | extentendly Dominated |
|  | |  | Sequence: **LEF** - **TNF** - Usual Treatment |  |  | 163.556 € | 317.627 € | Comparator |
|  | |  | Sequence: **TNF** - **LEF** - Usual Treatment |  |  | 297.151 € | 517.061 € | 1.155.314 € |
| **Wong** | | **2002** |  |  |  |  | **no discount** | **3% discount** |
| per QALY | |  | MTX |  |  |  | Comparator | Comparator |
|  | |  | INF/MTX |  |  |  | $26.800 | $30.500 |
| **Wu** | | **2012** | Sequence: Gold - LEF - CYC - MTX |  |  |  |  | Comparator |
| per QALY | |  | Sequence: **ADA** - Gold - LEF - CYC - MTX |  |  |  |  | $57.925 |
|  | |  | Sequence: **INF** - Gold - LEF - CYC - MTX |  |  |  |  | $26.813 |
|  | |  | Sequence: **ETA** - Gold - LEF - CYC - MTX |  |  |  |  | $77.394 |
|  | |  | Sequence: **ADA** - **RTX** - Gold - LEF - CYC - MTX |  |  |  |  | $51.001 |
|  | |  | Sequence: **INF** - **RTX** - Gold - LEF - CYC - MTX |  |  |  |  | $28.930 |
|  | |  | Sequence: **ETA** - **RTX** - Gold - LEF - CYC - MTX |  |  |  |  | $66.450 |
| **Yun** | | **2005** | NSAID |  |  |  |  | Comparator |
| per QALY | |  | NSAID/MIS |  |  |  |  | Extended Dominated |
|  | |  | NSAID/PPI |  |  |  |  | $56.751 |
|  | |  | COX-2 |  |  |  |  | $355.747 |
| **Yuan** | | **2010** | MTX |  |  |  |  | Comparator |
| per QALY | |  | ABA/MTX |  |  |  |  | $47.191 |
|  | |  | RTX/MTX |  |  |  |  | $54.891 |
