# Supplementary material for: Modeling rheumatoid arthritis using different techniques - a review of model construction and results
Source: Health Econ Rev. 2014 Sep 16;4:18. doi: 10.1186/s13561-014-0018-2 (PMC4502067; doi:10.1186/s13561-014-0018-2)
Supplement: Additional file 2: — Clinical Trial publications used for efficacy data in the CEA-models. [file 13561_2014_18_MOESM2_ESM.docx]

## Clinical Trial publications used for efficacy data in the CEA-models.

| Author | Year | Study | Number used | Citation |
| --- | --- | --- | --- | --- |
| Maini | 1999 | ATTRACT | 11 | [1] |
| Weinblatt | 1999 |  | 11 | [2] |
| Cohen | 2006 | REFLEX | 10 | [3] |
| Genovese | 2005 | ATTAIN | 9 | [4] |
| Moreland | 1999 |  | 9 | [5] |
| Genovese | 2008 |  | 7 | [6] |
| Keystone | 2004 |  | 7 | [7] |
| Bombardieri | 2007 |  | 6 | [8] |
| Keystone | 2007 |  | 5 | [9] |
| Smolen | 1999 |  | 5 | [10] |
| Strand | 1999 |  | 5 | [11] |
| Weinblatt | 2003 | ARMADA | 5 | [12] |
| Emery | 2000 |  | 4 | [13] |
| Lipsky | 2000 |  | 4 | [14] |
| Silverstein | 1995 |  | 4 | [15] |
| Bensen | 1999 |  | 3 | [16] |
| Fries | 1991 |  | 3 | [17] |
| Klareskog | 2004 | TEMPO | 3 | [18] |
| Lindqvist | 1999 |  | 3 | [19] |
| Maetzel | 1998 |  | 3 | [20] |
| SSATG database |  |  | 3 | - |
| Bathon | 2000 | ERA | 2 | [21] |
| Bergmann | 2010 |  | 2 | [22] |
| Bingham | 2009 |  | 2 | [23] |
| Bresnihan | 1998 |  | 2 | [24] |
| Burmester | 2007 |  | 2 | [25] |
| Crnkic | 2001 |  | 2 | [26] |
| Eberhardt | 1990 |  | 2 | [27] |
| Emery | 2005 |  | 2 | [28] |
| Emery | 1999 |  | 2 | [13] |
| Ericson | 1999 |  | 2 | [29] |
| Genovese | 2002 |  | 2 | [30] |
| Goldstein | 2001 |  | 2 | [31] |
| Goldstein | 2001a |  | 2 | [32] |
| Gutthann | 1996 |  | 2 | [33] |
| Hassett | 2008 |  | 2 | [34] |
| Hawkey | 1998 |  | 2 | [35] |
| Keystone | 2008 |  | 2 | [36] |
| Keystone | 2009 |  | 2 | [37] |
| Kremer | 2006 | AIM | 2 | [38] |
| Manoukian | 1996 |  | 2 | [39] |
| McKenna | 2001 |  | 2 | [40] |
| Miwa | 1997 |  | 2 | [41] |
| Nixon | 2007a |  | 2 | [42] |
| Simon | 1999 |  | 2 | [43] |
| Singh | 1998 |  | 2 | [44] |
| St. Clair | 2004 |  | 2 | [45] |
| STURE Registry | o.J. |  | 2 | - |
| Tugwell | 1995 |  | 2 | [46] |
| van de Putte | 2004 |  | 2 | [47] |
| Westhovens | 2006 |  | 2 | [48] |
| Alehata | 2006 |  | 1 | [49] |
| Anderson | 2000 |  | 1 | [50] |
| Bakowsky | 1999 |  | 1 | [51] |
| Bensen | 2000 |  | 1 | [52] |
| Bombardier | 2000 |  | 1 | [53] |
| Branicki | 1990 |  | 1 | [54] |
| Breedveld | 2005 |  | 1 | [55] |
| Browner | 1996 |  | 1 | [56] |
| Burke | 2001 |  | 1 | [57] |
| Cannon | 2000 |  | 1 | [58] |
| Chehata | 2001 |  | 1 | [59] |
| Chen | 2009 |  | 1 | [60] |
| Chrischilles | 1994 |  | 1 | [61] |
| Codreanu | 2003 |  | 1 | [62] |
| Cohen | 2002 |  | 1 | [63] |
| Cohen | 2001 |  | 1 | [64] |
| Day | 2000 |  | 1 | [65] |
| Duhamel | 1989 |  | 1 | [66] |
| Eberhardt | 1998 |  | 1 | [67] |
| Edwards | 2004 |  | 1 | [68] |
| Emery | 2010 |  | 1 | [69] |
| Felson | 1990 |  | 1 | [70] |
| Felson | 1992 |  | 1 | [71] |
| Felson | 1998 |  | 1 | [72] |
| Fisher | 1991 |  | 1 | [73] |
| Geborek | 2002 |  | 1 | [74] |
| Geborek | 2001 |  | 1 | [75] |
| Geborek | 2000 |  | 1 | [76] |
| Gerards | 2003 |  | 1 | [77] |
| Geusens | 2002 |  | 1 | [78] |
| Godil | 2000 |  | 1 | [79] |
| Goekoop-Ruiterman | 2005 |  | 1 | [80] |
| Goldkind | 2001 |  | 1 | [81] |
| Goldstein | 2000 |  | 1 | [82] |
| Hernandez-Diaz | 2000 |  | 1 | [83] |
| Hetland | 2010 |  | 1 | [84] |
| Holvoet | 1991 |  | 1 | [85] |
| Jones | 1995 |  | 1 | [86] |
| Kalden | 2001 |  | 1 | [87] |
| Karlsson | 2008 |  | 1 | [88] |
| Katschinski | 1994 |  | 1 | [89] |
| Kawai | 2002 |  | 1 | [90] |
| Kay | 2008 |  | 1 | [91] |
| Kievit | 2008 |  | 1 | [92] |
| Kievit | 2007 |  | 1 | [93] |
| Klein | 1993 |  | 1 | [94] |
| Koch | 1996 |  | 1 | [95] |
| Kremer | 2005 |  | 1 | [96] |
| Kristensen | 2008 |  | 1 | [97] |
| Kroot | 2000 |  | 1 | [98] |
| Kvien | 2002 |  | 1 | [99] |
| Laine | 1999 |  | 1 | [100] |
| Lan | 2004 |  | 1 | [101] |
| Landewe | 2006 |  | 1 | [102] |
| Langman | 1999 |  | 1 | [103] |
| Laszlo | 1998 |  | 1 | [104] |
| Loperfido | 1994 |  | 1 | [105] |
| McDougall | 1994 |  | 1 | [106] |
| Mease | 2010 |  | 1 | [107] |
| Moreland | 1997 |  | 1 | [108] |
| Munro | 1998 |  | 1 | [109] |
| NDB database (Wolfe 2005) |  |  | 1 | [110] |
| Nixon | 2007b | [1, 2, 4, 5, 7, 12, 18, 21, 45, 47, 63] | 1 | [111] |
| O'Dell | 1998 |  | 1 | [112] |
| Quinn | 2005 |  | 1 | [113] |
| Rahme | 2000 |  | 1 | [114] |
| Rau | 1991 |  | 1 | [115] |
| Riise | 2001 |  | 1 | [116] |
| Rostom | 2000 |  | 1 | [117] |
| Saag | 1994 |  | 1 | [118] |
| Saag | 2000 |  | 1 | [119] |
| Scheiman | 1998 |  | 1 | [120] |
| Scott | 2000 |  | 1 | [121] |
| Silverstein | 2000 |  | 1 | [122] |
| Singh | 2006 |  | 1 | [123] |
| Situnayake | 1987 |  | 1 | [124] |
| Smolen | 2009 |  | 1 | [125] |
| Smolen | 1995 |  | 1 | [126] |
| Smolen | 2008 |  | 1 | [127] |
| Symmons | 1998 |  | 1 | [128] |
| The HERA study group | 1995 | HERA | 1 | [129] |
| van Breedveld | 2004 | PREMIER | 1 | [130] |
| van der Heijde | 2006 | TEMPO | 1 | [131] |
| Verstraeten | 1986 |  | 1 | [132] |
| Walan | 1993 |  | 1 | [133] |
| Watson | 2003 |  | 1 | [134] |
| Welsing | 2004 |  | 1 | [135] |
| Wilcox | 1997 |  | 1 | [136] |
| Witter | 2001 |  | 1 | [137] |
| Wolfe | 1994 |  | 1 | [138] |
| Wong | 2001 |  | 1 | [139] |
| Yelin | 2002 |  | 1 | [140] |
| Zeidler | 1998 |  | 1 | [141] |
| Zimmermann | 1995 |  | 1 | [142] |
| Zink | 2006 |  | 1 | [143] |

**References**

1. Maini R, St Clair EW, Breedveld F et al. (1999) Infliximab (chimeric anti-tumour necrosis factor α monoclonal antibody) versus placebo in rheumatoid arthritis patients receiving concomitant methotrexate: a randomised phase III trial. The Lancet 354(9194): 1932–1939. doi: 10.1016/S0140-6736(99)05246-0

2. Weinblatt ME, Kremer JM, Bankhurst AD et al. (1999) A trial of etanercept, a recombinant tumor necrosis factor receptor:Fc fusion protein, in patients with rheumatoid arthritis receiving methotrexate. N Engl J Med 340(4): 253–259. doi: 10.1056/NEJM199901283400401

3. Cohen SB, Emery P, Greenwald MW et al. (2006) Rituximab for rheumatoid arthritis refractory to anti–tumor necrosis factor therapy: Results of a multicenter, randomized, double-blind, placebo-controlled, phase III trial evaluating primary efficacy and safety at twenty-four weeks. Arthritis Rheum 54(9): 2793–2806. doi: 10.1002/art.22025

4. Genovese MC, Becker J, Schiff M et al. (2005) Abatacept for Rheumatoid Arthritis Refractory to Tumor Necrosis Factor α Inhibition. N Engl J Med 353(11): 1114–1123. doi: 10.1056/NEJMoa050524

5. Moreland LW, Schiff MH, Baumgartner SW et al. (1999) Etanercept Therapy in Rheumatoid Arthritis. A Randomized, Controlled Trial. Ann Intern Med 130(6): 478–486

6. Genovese MC, Schiff M, Luggen M et al. (2008) Efficacy and safety of the selective co-stimulation modulator abatacept following 2 years of treatment in patients with rheumatoid arthritis and an inadequate response to anti-tumour necrosis factor therapy. Ann Rheum Dis 67(4): 547–554. doi: 10.1136/ard.2007.074773

7. Keystone EC, Kavanaugh AF, Sharp JT et al. (2004) Radiographic, clinical, and functional outcomes of treatment with adalimumab (a human anti-tumor necrosis factor monoclonal antibody) in patients with active rheumatoid arthritis receiving concomitant methotrexate therapy: a randomized, placebo-controlled, 52-week trial. Arthritis Rheum 50(5): 1400–1411. doi: 10.1002/art.20217

8. Bombardieri S, Ruiz AA, Fardellone P et al. (2007) Effectiveness of adalimumab for rheumatoid arthritis in patients with a history of TNF-antagonist therapy in clinical practice. Rheumatology 46(7): 1191–1199. doi: 10.1093/rheumatology/kem091

9. Keystone E, Fleischmann R, Emery P et al. (2007) Safety and efficacy of additional courses of rituximab in patients with active rheumatoid arthritis: An open-label extension analysis. Arthritis Rheum 56(12): 3896–3908. doi: 10.1002/art.23059

10. Smolen J, Kalden J, Scott D et al. (1999) Efficacy and safety of leflunomide compared with placebo and sulphasalazine in active rheumatoid arthritis: a double-blind, randomised, multicentre trial. The Lancet 353(9149): 259–266

11. Strand V JAMA Network | JAMA Internal Medicine | Nov 22, 1999. http://archinte.jamanetwork.com/issue.aspx?journalid=71&issueid=11954. Accessed 21 Jan 2013

12. Weinblatt ME, Keystone EC, Furst DE et al. (2003) Adalimumab, a fully human anti-tumor necrosis factor alpha monoclonal antibody, for the treatment of rheumatoid arthritis in patients taking concomitant methotrexate: the ARMADA trial. Arthritis Rheum 48(1): 35–45. doi: 10.1002/art.10697

13. Emery P, Breedveld FC, Lemmel EM et al. (2000) A comparison of the efficacy and safety of leflunomide and methotrexate for the treatment of rheumatoid arthritis. Rheumatology 39(6): 655–665. doi: 10.1093/rheumatology/39.6.655

14. Lipsky PE, van der Heijde DM, St. Clair EW et al. (2000) Infliximab and Methotrexate in the Treatment of Rheumatoid Arthritis. N Engl J Med 343(22): 1594–1602. doi: 10.1056/NEJM200011303432202

15. Silverstein FE, Graham DY, Senior JR et al. (1995) Misoprostol Reduces Serious Gastrointestinal Complications in Patients with Rheumatoid Arthritis Receiving Nonsteroidal Anti-Inflammatory DrugsA Randomized, Double-Blind, Placebo-Controlled Trial. Annals of Internal Medicine 123(4): 241–249. doi: 10.7326/0003-4819-123-4-199508150-00001

16. Bensen WG, Fiechtner JJ, McMillen JI et al. (1999) Treatment of Osteoarthritis With Celecoxib, a Cyclooxygenase-2 Inhibitor: A Randomized Controlled Trial. Mayo Clinic Proceedings 74(11): 1095–1105. doi: 10.4065/74.11.1095

17. Fries JF, Williams CA, Bloch DA et al. (1991) Nonsteroidal anti-inflammatory drug-associated gastropathy: Incidence and risk factor models. The American Journal of Medicine 91(3): 213–222. doi: 10.1016/0002-9343(91)90118-H

18. Klareskog L, van der Heijde DMFM, Jager JP de et al. (2004) Therapeutic effect of the combination of etanercept and methotrexate compared with each treatment alone in patients with rheumatoid arthritis: double-blind randomised controlled trial. Lancet 363(9410): 675–681

19. Lindqvist E, Eberhardt K (1999) Mortality in rheumatoid arthritis patients with disease onset in the 1980s. Annals of the Rheumatic Diseases 58(1): 11–14. doi: 10.1136/ard.58.1.11

20. Maetzel A, Ferraz MB, Bombardier C (1998) The cost-effectiveness of misoprostol in preventing serious gastrointestinal events associated with the use of nonsteroidal antiinflammatory drugs. Arthritis & Rheumatism 41(1): 16–25. doi: 10.1002/1529-0131(199801)41:1<16:AID-ART3>3.0.CO;2-4

21. Bathon JM, Martin RW, Fleischmann RM et al. (2000) A Comparison of Etanercept and Methotrexate in Patients with Early Rheumatoid Arthritis. N Engl J Med 343(22): 1586–1593. doi: 10.1056/NEJM200011303432201

22. Bergman GJD, Hochberg MC, Boers M et al. (2010) Indirect Comparison of Tocilizumab and Other Biologic Agents in Patients with Rheumatoid Arthritis and Inadequate Response to Disease-Modifying Antirheumatic Drugs. Semin Arthritis Rheum 39(6): 425–441

23. Bingham CO, Looney RJ, Deodhar A et al. (2010) Immunization responses in rheumatoid arthritis patients treated with rituximab: Results from a controlled clinical trial. Arthritis Rheum 62(1): 64–74. doi: 10.1002/art.25034

24. Bresnihan B AJCMMDZEP (1998) Treatment of rheumatoid arthritis with recombinant human interleukin-1 receptor antagonist. Arthritis Rheum 41(12): 2196–2204

25. Burmester GR, Mariette X, Montecucco C et al. (2007) Adalimumab alone and in combination with disease-modifying antirheumatic drugs for the treatment of rheumatoid arthritis in clinical practice: the Research in Active Rheumatoid Arthritis (ReAct) trial. Ann Rheum Dis 66(6): 732–739. doi: 10.1136/ard.2006.066761

26. Crnkic M, Petersson I, Saxne T et al. (2001) Infiximab, etanercept and leflunomide in rheumatoid arthritis. Clinical experience in southern Sweden [abstract]. BSR conference proceedings: 231a

27. Eberhardt KB, Rydgren LC, Pettersson H et al. (1990) Early rheumatoid arthritis —onset, course, and outcome over 2 years. Rheumatol Int 10(4): 135-142. doi: 10.1007/BF02274837

28. Emery P, Fleischmann R, Filipowicz-Sosnowska A et al. (2006) The efficacy and safety of rituximab in patients with active rheumatoid arthritis despite methotrexate treatment: Results of a phase IIB randomized, double-blind, placebo-controlled, dose-ranging trial. Arthritis Rheum 54(5): 1390–1400. doi: 10.1002/art.21778

29. Ericson M WJobotE (1999) A double-blind, placebo controlled study of the efficacy and safety of four different doses of etanercept in patients with rheumatoid arthritis. In: ACR/ARHP scientific abstracts. John Wiley & Sons, Inc, pp S82

30. Genovese MC, Bathon JM, Martin RW et al. (2002) Etanercept versus methotrexate in patients with early rheumatoid arthritis: Two-year radiographic and clinical outcomes. Arthritis Rheum 46(6): 1443–1450. doi: 10.1002/art.10308

31. Goldstein JL, Agrawal N, Eisen GM et al. (2001) Significantly improved upper gastrointestinal (UGI) telerability with celecoxib, a COX-2 specific inhibitor, compared with conventional NSAIDs. The SUCCESS I trial. Gastroenterology 120(5): A105

32. Goldstein JL, Eisen GM, Stenson W et al. (2001) Significant reduction in serious upper gastrointestinal (UGI) events with celecoxib, a COX-2 specific inhibitor, compared with conventional NSAIDs. The SUCCESS I trial. Gastroenterology 120(5): A105

33. Gutthann S, Rodríguez L, Raiford DS et al. (1996) NOnsteroidal anti-inflammatory drugs and the risk of hospitalization for acute renal failure. Archives of Internal Medicine 156(21): 2433–2439. doi: 10.1001/archinte.1996.00440200041005

34. Hassett AL, Li T, Buyske S et al. (2008) The multi-faceted assessment of independence in patients with rheumatoid arthritis: preliminary validation from the ATTAIN study*. Curr Med Res Opin 24(5): 1443–1453. doi: 10.1185/030079908X297376

35. Hawkey CJ, Karrasch JA, Szczepañski L et al. (1998) Omeprazole Compared with Misoprostol for Ulcers Associated with Nonsteroidal Antiinflammatory Drugs. New England Journal of Medicine 338(11): 727–734. doi: 10.1056/NEJM199803123381105

36. Keystone E, van der Heijde D, Mason D et al. (2008) Certolizumab pegol plus methotrexate is significantly more effective than placebo plus methotrexate in active rheumatoid arthritis: Findings of a fifty-two–week, phase III, multicenter, randomized, double-blind, placebo-controlled, parallel-group study. Arthritis Rheum 58(11): 3319–3329. doi: 10.1002/art.23964

37. Keystone EC, Genovese MC, Klareskog L et al. (2009) Golimumab, a human antibody to tumour necrosis factor α given by monthly subcutaneous injections, in active rheumatoid arthritis despite methotrexate therapy: the GO-FORWARD Study. Ann Rheum Dis 68(6): 789–796. doi: 10.1136/ard.2008.099010

38. Kremer JM, Genant HK, Moreland LW et al. (2006) Effects of Abatacept in Patients with Methotrexate-Resistant Active Rheumatoid ArthritisA Randomized Trial. Ann Intern Med 144(12): 865–876

39. Manoukian A, Carson J (1996) Nonsteroidal Anti-Inflammatory Drug-Induced Hepatic Disorders. Drug-Safety 15(1): 64-71. doi: 10.2165/00002018-199615010-00005

40. F. McKenna DBHWCWJBLGSG (2001) Celecoxib versus diclofenac in the management of osteoarthritis of the knee: A placebo-controlled, randomised, double-blind comparison. Scandinavian Journal of Rheumatology 30(1): 11–18. doi: 10.1080/030097401750065265

41. Miwa LJ, Jones JK, Pathiyal A et al. (1997) Value of epidemiologic studies in determining the true incidence of adverse events: The nonsteroidal anti-inflammatory drug story. Archives of Internal Medicine 157(18): 2129–2136. doi: 10.1001/archinte.1997.00440390131016

42. Nixon R, Bansback N, Brennan A (2007) The efficacy of inhibiting tumour necrosis factor α and interleukin 1 in patients with rheumatoid arthritis: a meta-analysis and adjusted indirect comparisons. Rheumatology 46(7): 1140–1147. doi: 10.1093/rheumatology/kem072

43. Simon LS, Weaver AL, Graham DY et al. (1999) Anti-inflammatory and upper gastrointestinal effects of celecoxib in rheumatoid arthritis: A randomized controlled trial. JAMA 282(20): 1921–1928. doi: 10.1001/jama.282.20.1921

44. Singh G, Rosen Ramey D (1998) NSAID induced gastrointestinal complications: the ARAMIS perspective--1997. Arthritis, Rheumatism, and Aging Medical Information System. J Rheumatol Suppl 51: 8-16

45. St. Clair EW, van der Heijde DMFM, Smolen JS et al. (2004) Combination of infliximab and methotrexate therapy for early rheumatoid arthritis: A randomized, controlled trial. Arthritis Rheum 50(11): 3432–3443. doi: 10.1002/art.20568

46. Tugwell P, Pincus T, Yocum D et al. (1995) Combination Therapy with Cyclosporine and Methotrexate in Severe Rheumatoid Arthritis. N Engl J Med 333(3): 137–142. doi: 10.1056/NEJM199507203330301

47. van de Putte L, Atkins C, Malaise M, Sany J, Russell A, P L C M van Riel, Settas L, Bijlsma J, Todesco S, Dougados M, Nash P, Emery P, Walter N, Kaul M, Fischkoff S, Kupper H (2004) Efficacy and safety of adalimumab as monotherapy in patients with rheumatoid arthritis for whom previous disease modifying antirheumatic drug treatment has failed. http://www.ncbi.nlm.nih.gov/pmc/articles/PMC1755008/. Accessed 21 Jan 2013

48. Westhovens R, Yocum D, Han J et al. (2006) The safety of infliximab, combined with background treatments, among patients with rheumatoid arthritis and various comorbidities: A large, randomized, placebo-controlled trial. Arthritis Rheum 54(4): 1075–1086. doi: 10.1002/art.21734

49. Aletaha D, Smolen J, Ward MM (2006) Measuring function in rheumatoid arthritis: Identifying reversible and irreversible components. Arthritis Rheum 54(9): 2784–2792. doi: 10.1002/art.22052

50. Anderson J, Wells G, Verhoeven A et al. (2000) Factors predicting response to treatment in rheumatoid arthritis. the importance of disease duration. Arthritis Rheum 43: 22–29

51. Bakowsky V, Hanly J (1999) Complications of nonsteroidal antiinflammatory drug gastropathy and use of gastric cytoprotection:experience in a tertiary health care center. J Rheumatol 26: 1557–1563

52. Bensen WG, Zhao SZ, Burke TA et al. (2000) Upper gastrointestinal tolerability of celecoxib, a COX-2 specific inhibitor, compared to naproxen and placebo. J Rheumatol 27(8): 1876-1883

53. Bombardier C, Laine L, Reicin A et al. (2000) Comparison of Upper Gastrointestinal Toxicity of Rofecoxib and Naproxen in Patients with Rheumatoid Arthritis. New England Journal of Medicine 343(21): 1520–1528. doi: 10.1056/NEJM200011233432103

54. Branicki F, Coleman SY, Fok P et al. (1990) Bleeding peptic ulcer: A prospective evaluation of risk factors for rebleeding and mortality. World J. Surg. 14(2): 262-269. doi: 10.1007/BF01664889

55. Breedveld FC, Weisman MH, Kavanaugh A et al. (2005 Jun 8-11) The efficacy and safety of adalimumab (Humira(c)) plus methotrexate vs. adalimumab alone or methotrexate alone in the early treatment of rheumatoid arthritis (RA). 1 and 2 year results of the PREMIER study [OP0013]. The Annual European Congress of Rheumatology, Vienna

56. Browner WS, Pressman AR, Nevitt MC et al. (1996) Mortality following fractures in older women: The study of osteoporotic fractures. Archives of Internal Medicine 156(14): 1521–1525. doi: 10.1001/archinte.1996.00440130053006

57. Burke T, Zabinski R, Pettitt D et al. (2001) A Framework for Evaluating the Clinical Consequences of Initial Therapy with NSAIDs, NSAIDs plus Gastroprotective Agents, or Celecoxib in the Treatment of Arthritis. Pharmacoeconomics 19(1): 33-47. doi: 10.2165/00019053-200119001-00003

58. Cannon GW, Caldwell JR, Holt P et al. (2000) Rofecoxib, a specific inhibitor of cyclooxygenase 2, with clinical efficacy comparable with that of diclofenac sodium: Results of a one-year, randomized, clinical trial in patients with osteoarthritis of the knee and hip. Arthritis & Rheumatism 43(5): 978–987. doi: 10.1002/1529-0131(200005)43:5<978:AID-ANR4>3.0.CO;2-0

59. Chehata JC, Hassell AB, Clarke SA et al. (2001) Mortality in rheumatoid arthritis: relationship to single and composite measures of disease activity. Rheumatology 40(4): 447–452. doi: 10.1093/rheumatology/40.4.447

60. Chen Y, Yan W, Geczy C et al. (2009) Serum levels of soluble receptor for advanced glycation end products and of S100 proteins are associated with inflammatory, autoantibody, and classical risk markers of joint and vascular damage in rheumatoid arthritis. Arthritis Res Ther 11(2): 1-11. doi: 10.1186/ar2645

61. Chrischilles E, Shireman T, Wallace R (1994) Costs and health effects of osteoporotic fractures. Bone 15(4): 377–386. doi: 10.1016/8756-3282(94)90813-3

62. Codreanu C CBFUGMGPKTea (2003) Double-blind comparison of etanercept and sulfasalazine, alone and combined in active RA patients [abstract THU0120]. In: EULAR

63. Cohen S, Hurd E, Cush J et al. (2002) Treatment of rheumatoid arthritis with anakinra, a recombinant human interleukin-1 receptor antagonist, in combination with methotrexate: Results of a twenty-four–week, multicenter, randomized, double-blind, placebo-controlled trial. Arthritis Rheum 46(3): 614–624. doi: 10.1002/art.10141

64. Cohen S, Cannon GW, Schiff M et al. (2001) Two-year, blinded, randomized, controlled trial of treatment of active rheumatoid arthritis with leflunomide compared with methotrexate. Arthritis Rheum 44(9): 1984–1992. doi: 10.1002/1529-0131(200109)44:9<1984:AID-ART346>3.0.CO;2-B

65. Day R, Morrison B, Luza A et al. (2000) A randomized trial of the efficacy and tolerability of the cox-2 inhibitor rofecoxib vs ibuprofen in patients with osteoarthritis. Archives of Internal Medicine 160(12): 1781–1787. doi: 10.1001/archinte.160.12.1781

66. Duhamel C, Czernichow P, Dechelotte P et al. (1989) Upper gastrointestinal hemorrhage caused by anti-inflammatory agents. Gastroenterol Clin Biol 13(3): 239-244

67. Eberhardt K, Fex E (1998) Clinical course and remission rate in patients with early rheumatoid arthritis: relationship to outcome after 5 years. Rheumatology 37(12): 1324–1329. doi: 10.1093/rheumatology/37.12.1324

68. Edwards JC, Szczepański L, Szechiński J et al. (2004) Efficacy of B-Cell–Targeted Therapy with Rituximab in Patients with Rheumatoid Arthritis. N Engl J Med 350(25): 2572–2581. doi: 10.1056/NEJMoa032534

69. Emery P, Breedveld F, van der Heijde D et al. (2010) Two-year clinical and radiographic results with combination etanercept–methotrexate therapy versus monotherapy in early rheumatoid arthritis: A two-year, double-blind, randomized study. Arthritis Rheum 62(3): 674–682. doi: 10.1002/art.27268

70. Felson DT, Anderson JJ, Meenan RF (1990) The comparative efficacy and toxicity of second-line drugs in rheumatoid arthritis results of two metaanalyses. Arthritis Rheum 33(10): 1449–1461. doi: 10.1002/art.1780331001

71. Felson DT, Anderson JJ, Meenan RF (1992) Use of short-term efficacy/toxicity tradeoffs to select second-line drugs in rheumatoid arthritis. A metaanalysis of published clinical trials. Arthritis Rheum 35(10): 1117–1125. doi: 10.1002/art.1780351003

72. Felson DT, Anderson JJ, Lange MLM et al. (1998) Should imporvement in rheumatoid arthritis clinical trials be defined as fifty percent or seventy percent improvement in core set measures, rather than twenty percent? Arthritis Rheum 41(9): 1564–1570. doi: 10.1002/1529-0131(199809)41:9<1564:AID-ART6>3.0.CO;2-M

73. Fisher ES, Baron JA, Malenka DJ et al. (1991) Hip Fracture Incidence and Mortality in New England. Epidemiology 2(2): 116–122. doi: 10.2307/25759862

74. Geborek P, Crnkic M, Petersson I, Saxne T (2002) Etanercept, infliximab, and leflunomide in established rheumatoid arthritis: clinical experience using a structured follow up programme in southern Sweden. http://www.ncbi.nlm.nih.gov/pmc/articles/PMC1754224/. Accessed 21 Jan 2013

75. Geborek P, Crnkic M, Petersson I et al. (2001) Efficacy of etanercept, infliximab and leflunomide in rheumatoid arthritis (RA). Experience using a clinical protocol on a regional basis. 40(Supplement 1)

76. Geborek P, Saxne T (2000) Clinical protocol for monitoring of targeted therapies in rheumatoid arthritis. Rheumatology 39(10): 1159–1161. doi: 10.1093/rheumatology/39.10.1159

77. Gerards AH, Landewé RBM, Prins APA et al. (2003) Cyclosporin A monotherapy versus cyclosporin A and methotrexate combination therapy in patients with early rheumatoid arthritis: a double blind randomised placebo controlled trial. Ann Rheum Dis 62(4): 291–296. doi: 10.1136/ard.62.4.291

78. Geusens PP, Truitt K, Sfikakis P et al. (2002) A placebo and active comparator-controlled trial of rofecoxib for the treatment of rheumatoid arthritis. Scandinavian Journal of Rheumatology 31(4): 230–238. doi: 10.1080/030097402320318431

79. Godil A, DeGuzman L, Schilling R, III et al. (2000) Recent nonsteroidal anti-inflammatory drug use increases the risk of early recurrence of bleeding in patients presenting with bleeding ulcer. Gastrointest Endosc 51: 146–151

80. Goekoop-Ruiterman YPM, Vries-Bouwstra JK de, Allaart CF et al. (2005) Clinical and radiographic outcomes of four different treatment strategies in patients with early rheumatoid arthritis (the BeSt study): A randomized, controlled trial. Arthritis Rheum 52(11): 3381–3390. doi: 10.1002/art.21405

81. Goldkind L (2001) Medical officer's advisory comitee GI briefing document division of anti-inflammatory, analgesic and ophtamologic drug products: HFD-550. NDA 21-042: 1–56

82. Goldstein JL, Silverstein FE, Agrawal NM et al. (2000) Reduced risk of upper gastrointestinal ulcer complications with celecoxib, a novel COX-2 inhibitor. The American Journal of Gastroenterology 95(7): 1681–1690. doi: 10.1016/S0002-9270(00)00986-2

83. Hernández-Díaz S, Rodríguez L (2000) Association between nonsteroidal anti-inflammatory drugs and upper gastrointestinal tract bleeding/perforation: An overview of epidemiologic studies published in the 1990s. Archives of Internal Medicine 160(14): 2093–2099. doi: 10.1001/archinte.160.14.2093

84. Hetland ML, Christensen IJ, Tarp U et al. (2010) Direct comparison of treatment responses, remission rates, and drug adherence in patients with rheumatoid arthritis treated with adalimumab, etanercept, or infliximab: Results from eight years of surveillance of clinical practice in the nationwide Danish DANBIO registry. Arthritis & Rheumatism 62(1): 22–32. doi: 10.1002/art.27227

85. Holvoet J, Terriere L, van Hee W et al. (1991) Relation of upper gastrointestinal bleeding to non-steroidal anti-inflammatory drugs and aspirin: a case-control study. Gut 32(7): 730–734. doi: 10.1136/gut.32.7.730

86. Jones RH, Tait CL (1995) Gastrointestinal side-effects of NSAIDs in the community. Br J Clin Pract 49(2): 67-70

87. Kalden JR, Scott DL, Smolen JS et al. (2001) Improved functional ability in patients with rheumatoid arthritis--longterm treatment with leflunomide versus sulfasalazine. European Leflunomide Study Group. J Rheumatol 28(9): 1983–1991

88. Karlsson JA, Kristensen LE, Kapetanovic MC et al. (2008) Treatment response to a second or third TNF-inhibitor in RA: results from the South Swedish Arthritis Treatment Group Register. Rheumatology 47(4): 507–513. doi: 10.1093/rheumatology/ken034

89. Katschinski B, Logan R, Davies J et al. (1994) Prognostic factors in upper gastrointestinal bleeding. Digest Dis Sci 39(4): 706-712. doi: 10.1007/BF02087411

90. Kawai S, Kato T, Matsuda T (2002) Quality of life measures as clincal assessment of improvement in rheumatoid arthritis. Recent Adv Clin Pharmacol 23: 47–51

91. Kay J, Matteson EL, Dasgupta B et al. (2008) Golimumab in patients with active rheumatoid arthritis despite treatment with methotrexate: A randomized, double-blind, placebo-controlled, dose-ranging study. Arthritis Rheum 58(4): 964–975. doi: 10.1002/art.23383

92. Kievit W, Adang EM, Fransen J et al. (2008) The effectiveness and medication costs of three anti-tumour necrosis factor α agents in the treatment of rheumatoid arthritis from prospective clinical practice data. Ann Rheum Dis 67(9): 1229–1234. doi: 10.1136/ard.2007.083675

93. Kievit W, Fransen J, Oerlemans AJM et al. (2007) The efficacy of anti-TNF in rheumatoid arthritis, a comparison between randomised controlled trials and clinical practice. Annals of the Rheumatic Diseases 66(11): 1473–1478. doi: 10.1136/ard.2007.072447

94. Klein W, Krevsky B, Klepper L et al. (1993) Nonsteroidal antiinflammatory drugs and upper gastrointestinal hemorrhage in an urban hospital. Digest Dis Sci 38(11): 2049-2055. doi: 10.1007/BF01297084

95. Koch M, Dezi A, Ferrario F et al. (1996) Prevention of nonsteroidal anti-inflammatory drug-induced gastrointestinal mucosal injury. A meta-analysis of randomized controlled clinical trials. Arch Intern Med 156(20): 2321-2332

96. Kremer JM, Dougados M, Emery P et al. (2005) Treatment of rheumatoid arthritis with the selective costimulation modulator abatacept: Twelve-month results of a phase iib, double-blind, randomized, placebo-controlled trial. Arthritis Rheum 52(8): 2263–2271. doi: 10.1002/art.21201

97. Kristensen LE, Kapetanovic MC, Gülfe A et al. (2008) Predictors of response to anti-TNF therapy according to ACR and EULAR criteria in patients with established RA: results from the South Swedish Arthritis Treatment Group Register. Rheumatology 47(4): 495–499. doi: 10.1093/rheumatology/ken002

98. Kroot EJA, van Leeuwen MA, van Rijswijk MH et al. (2000) No increased mortality in patients with rheumatoid arthritis: up to 10 years of follow up from disease onset. Annals of the Rheumatic Diseases 59(12): 954–958. doi: 10.1136/ard.59.12.954

99. Kvien TK ZHHPWFFOHIea (2002) Long term efficacy and safety of cyclosporin versus parental gold in early rheumatoid arthritis: a three year study of radiographic progression, renal function, and arterial hypertension. Ann Rheum Dis 61(6)

100. Laine L, Harper S, Simon T et al. (1999) A randomized trial comparing the effect of rofecoxib, a cyclooxygenase 2–specific inhibitor, with that of ibuprofen on the gastroduodenal mucosa of patients with osteoarthritis. Gastroenterology 117(4): 776–783. doi: 10.1016/S0016-5085(99)70334-3

101. LAN Joung-Liang, CHOU Show-Jan, CEN Der-Yuan et al. (2004) A comparative study of etanercept plus methotrexate and methotrexate alone in Taiwanese patients with active rheumatoid arthritis : A 12-week, double-blind, randomized, placebo-controlled study. Journal of the Formosan Medical Association 103(8): 618–623

102. Landewé R, van der Heijde D, Klareskog L et al. (2006) Disconnect between inflammation and joint destruction after treatment with etanercept plus methotrexate: Results from the trial of etanercept and methotrexate with radiographic and patient outcomes. Arthritis Rheum 54(10): 3119–3125. doi: 10.1002/art.22143

103. Langman MJ, Jensen DM, Watson DJ et al. (1999) ADverse upper gastrointestinal effects of rofecoxib compared with nsaids. JAMA 282(20): 1929–1933. doi: 10.1001/jama.282.20.1929

104. Laszlo A, Kelly JP, Kaufman DE et al. (1998) Clinical aspects of upper gastrointestinal bleeding associated with the use of nonsteroidal antiinflammatory drugs. Am J Gastroenterol 93(5): 721–725

105. Loperfido S, Monica F, Maifreni L et al. (1994) Bleeding peptic ulcer occurring in hospitalized patients. Digest Dis Sci 39(4): 698-705. doi: 10.1007/BF02087410

106. McDougall R, Sibley J, Haga M et al. (1994) Outcome in patients with rheumatoid arthritis receiving prednisone compared to matched controls. J Rheumatol 21(7): 1207-1213

107. Mease PJ, Cohen S, GAYLIS NB et al. (2010) Efficacy and Safety of Retreatment in Patients with Rheumatoid Arthritis with Previous Inadequate Response to Tumor Necrosis Factor Inhibitors: Results from the SUNRISE Trial. The Journal of Rheumatology 37(5): 917–927. doi: 10.3899/jrheum.090442

108. Moreland LW, Baumgartner SW, Schiff MH et al. (1997) Treatment of rheumatoid arthritis with a recombinant human tumor necrosis factor receptor (p75)-Fc fusion protein. N Engl J Med 337(3): 141–147. doi: 10.1056/NEJM199707173370301

109. Munro R, Hampson R, McEntegart A et al. (1998) Improved functional outcome in patients with early rheumatoid arthritis treated with intramuscular gold: results of a five year prospective study. Ann Rheum Dis 57(2): 88–93. doi: 10.1136/ard.57.2.88

110. Wolfe F, Michaud K (2005) A brief introduction to the National Data Bank for Rheumatic Diseases. Clin Exp Rheumatol 23(Supplement 2): S168-S171

111. Nixon RM, Bansback N, Brennan A (2007) Using mixed treatment comparisons and meta-regression to perform indirect comparisons to estimate the efficacy of biologic treatments in rheumatoid arthritis. Statist. Med. 26(6): 1237–1254. doi: 10.1002/sim.2624

112. O'Dell JR (1998) TRIPLE THERAPY WITH METHOTREXATE, SULFASALAZINE, AND HYDROXYCHLOROQUINE IN PATIENTS WITH RHEUMATOID ARTHRITIS. Rheumatic Disease Clinics of North America 24(3): 465–477. doi: 10.1016/S0889-857X(05)70020-X

113. Quinn MA, Conaghan PG, O'Connor PJ et al. (2005) Very early treatment with infliximab in addition to methotrexate in early, poor-prognosis rheumatoid arthritis reduces magnetic resonance imaging evidence of synovitis and damage, with sustained benefit after infliximab withdrawal: Results from a twelve-month randomized, double-blind, placebo-controlled trial. Arthritis Rheum 52(1): 27–35. doi: 10.1002/art.20712

114. Rahme E, Joseph L, Kong SX et al. (2000) Gastrointestinal health care resource use and costs associated with nonsteroidal antiinflammatory drugs versus acetaminophen: Retrospective cohort study of an elderly population. Arthritis & Rheumatism 43(4): 917–924. doi: 10.1002/1529-0131(200004)43:4<917:AID-ANR25>3.0.CO;2-F

115. Rau R, Herborn G, Karger T et al. (1991) A double-blind comparison of parenteral methotrexate and parenteral gold in the treatment of early erosive rheumatoid arthritis: An interim report after 12 months. Semin Arthritis Rheum 21(Supplement): 13–20

116. Riise T, Jacobsen BK, Gran JT et al. (2001) Total Mortality is Increased in Rheumatoid Arthritis. A 17-Year Prospective Study. Clin Rheumatol 20(2): 123-127. doi: 10.1007/PL00011191

117. Rostom A, Wells G, Tugwell P et al. (2000) The prevention of chronic NSAID induced upper gastrointestinal toxicity: a Cochrane collaboration metaanalysis of randomized controlled trials. J Rheumatol 27(9): 2203-2214

118. Saag KG, Koehnke R, Caldwell JR et al. (1994) Low dose long-term corticosteroid therapy in rheumatoid arthritis: An analysis of serious adverse events. The American Journal of Medicine 96(2): 115–123. doi: 10.1016/0002-9343(94)90131-7

119. Saag KG, van der Heijde DM, Fisher C et al. (2000) Rofecoxib, a New Cyclooxygenase 2 Inhibitor, Shows Sustained Efficacy, Comparable With Other Nonsteroidal Anti-inflammatory Drugs. A 6-Week and a 1-Year Trial in Patients With Osteoarthritis. Archives of Family Medicine 9(10): 1124–1134

120. Scheiman J, Arbor A, Isenberg J (1998) Agents used in the prevention and treatment of nonsteroidal anti-inflammatory drug-associated symptoms and ulcers. The American Journal of Medicine 105(5, Supplement 1): 32S. doi: 10.1016/S0002-9343(98)00279-4

121. Scott DL, Pugner K, Kaarela K et al. (2000) The links between joint damage and disability in rheumatoid arthritis. Rheumatology 39(2): 122–132. doi: 10.1093/rheumatology/39.2.122

122. Silverstein FE, Faich G, Goldstein JL et al. (2000) Gastrointestinal toxicity with celecoxib vs nonsteroidal anti-inflammatory drugs for osteoarthritis and rheumatoid arthritis: The class study: a randomized controlled trial. JAMA 284(10): 1247–1255. doi: 10.1001/jama.284.10.1247

123. Singh G, Fort JG, Goldstein JL et al. (2006) Celecoxib Versus Naproxen and Diclofenac in Osteoarthritis Patients: SUCCESS-I Study. The American Journal of Medicine 119(3): 255–266. doi: 10.1016/j.amjmed.2005.09.054

124. Situnayake RD, Grindulis KA, McConkey B (1987) Long-term treatment of rheumatoid arthritis with sulphasalazine, gold, or penicillamine: a comparison using life-table methods. Ann Rheum Dis 46(3): 177–183. doi: 10.1136/ard.46.3.177

125. Smolen JS, Kay J, Doyle MK et al. (2009) Golimumab in patients with active rheumatoid arthritis after treatment with tumour necrosis factor ? inhibitors (GO-AFTER study): a multicentre, randomised, double-blind, placebo-controlled, phase III trial. Lancet 374(9685): 210–221

126. Smolen JS, Eberl G, Breedveld FC et al. (1995) Validity and reliability of the twenty-eight-joint count for the assessment of rheumatoid arthritis activity. Arthritis & Rheumatism 38(1): 38–43. doi: 10.1002/art.1780380106

127. Smolen JS, Beaulieu A, Rubbert-Roth A et al. (2008) Effect of interleukin-6 receptor inhibition with tocilizumab in patients with rheumatoid arthritis (OPTION study): a double-blind, placebo-controlled, randomised trial. The Lancet 371(9617): 987–997. doi: 10.1016/S0140-6736(08)60453-5

128. Symmons DP, Jones MA, Scott DL et al. (1998) Longterm mortality outcome in patients with rheumatoid arthritis: early presenters continue to do well. J Rheumatol 25(6): 1072-1077

129. The HERA study group (1995) A randomized trial of hydroxychloroquine in early rheumatoid arthritis: The HERA study. Am J Med 98(2): 156–168

130. van Breedveld FC KACSPKVRPJ (2004) Early treatment of rheumatoid arthritis (RA) with adalimumab (HUMIRA®) plus methotrexate vs. adalimumab alone or methotrexate alone.The PREMIER study [abstract L5]. Arthritis Rheum 50: 4096–4097

131. van der Heijde D, Klareskog L, Rodriguez-Valverde V et al. (2006) Comparison of etanercept and methotrexate, alone and combined, in the treatment of rheumatoid arthritis: Two-year clinical and radiographic results from the TEMPO study, a double-blind, randomized trial. Arthritis Rheum 54(4): 1063–1074. doi: 10.1002/art.21655

132. Verstraeten A, Dequeker J (1986) Vertebral and peripheral bone mineral content and fracture incidence in postmenopausal patients with rheumatoid arthritis: effect of low dose corticosteroids. Annals of the Rheumatic Diseases 45(10): 852–857. doi: 10.1136/ard.45.10.852

133. Walan A, Wahlqvist P (1999) Pharmacoeconomic aspects of non-steroidal anti-inflammatory drug gastropathy. Ital J Gastroenterol Hepatol 31 Suppl 1: S79-88

134. Watson D, Yu C, Bolognese J et al. (2003) Improved upper-GI safety with etoricoxib compared with non-selective cyclooxygenase inhibitors (NSAIDs). Arthritis Rheum 48(Supplement 9): S72

135. Welsing PMJ, van Riel PLCM (2004) The Nijmegen inception cohort of early rheumatoid arthritis. J Rheumatol 69: 14–21

136. Wilcox CM, Clark WS (1997) Association of Nonsteroidal Antiinflammatory Drugs with Outcome in Upper and Lower Gastrointestinal Bleeding. Dig Dis Sci 42(5): 985-989. doi: 10.1023/A:1018880818217

137. Witter J (2001) Medical officer review: sNDA 20 998, Rockville (MD)

138. Wolfe F, Mitchell DM, Sibley JT et al. (1994) The mortality of rheumatoid arthritis. Arthritis & Rheumatism 37(4): 481–494. doi: 10.1002/art.1780370408

139. Wong JB, Ramey DR, Singh G (2001) Long-term morbidity, mortality, and economics of rheumatoid arthritis. Arthritis Rheum 44(12): 2746–2749. doi: 10.1002/1529-0131(200112)44:12<2746:AID-ART461>3.0.CO;2-Z

140. Yelin E, Trupin L, Wong B et al. (2002) The impact of functional status and change in functional status on mortality over 18 years among persons with rheumatoid arthritis. The Journal of Rheumatology 29(9): 1851–1857

141. Zeidler HK, Kvien TK, Hannonen P et al. (1998) Progression of joint damage in early active severe rheumatoid arthritis during 18 months of treatment: comparison of low-dose cyclosporin and parenteral gold. Br J Rheumatol 37(8): 874–882

142. Zimmerman J, Siguencia J, Tsvang E et al. (1995) Predictors of Mortality in Patients Admitted to Hospital for Acute Upper Gastrointestinal Hemorrhage. Scandinavian Journal of Gastroenterology 30(4): 327–331. doi: 10.3109/00365529509093285

143. Zink A, Strangfeld A, Schneider M et al. (2006) Effectiveness of tumor necrosis factor inhibitors in rheumatoid arthritis in an observational cohort study: Comparison of patients according to their eligibility for major randomized clinical trials. Arthritis & Rheumatism 54(11): 3399–3407. doi: 10.1002/art.22193
